# From molecular to cellular form: modeling the first major transition during the arising of life

**DOI:** 10.1186/s12862-019-1412-5

**Published:** 2019-04-03

**Authors:** Shaolin Yin, Yong Chen, Chunwu Yu, Wentao Ma

**Affiliations:** 1 Hubei Key Laboratory of Cell Homeostasis, College of Life Sciences, Wuhan University, Wuhan, 430072 People’s Republic of China; 2 College of Computer Sciences, Wuhan University, Wuhan, 430072 People’s Republic of China

**Keywords:** The origin of life, Prebiotic evolution, In silico simulation, Evolutionary dynamics

## Abstract

**Background:**

It has long been suggested that Darwinian evolution may have started at the molecular level and subsequently proceeded to a level with membrane boundary, i.e., of protocells. The transformation has been referred to as “the first major transition leading to life”. However, so far, we actually have little knowledge about the relevant evolutionary mechanisms – and even about the plausibility – of such a transition. Here, based upon the scenario of the RNA world, we performed a computer simulation study to address this issue.

**Results:**

First, it was shown that at the molecular level, after the spread of one ribozyme (RNA replicase), another ribozyme (nucleotide synthetase) may emerge naturally in the system, and the two ribozymes would cooperate to spread in the naked scene. Then, when empty vesicles absorb the two ribozymes via “cytophagy”, the resulting protocells may spread in the system and substitute the naked ribozymes. As for the driven power of such a transition, it was demonstrated that the membrane boundary’s roles to ensure the cooperation between the two ribozymes and to prevent invasion of parasites are important. Beyond that, remarkably, it was found that another two factors may also have been significant: a possibly higher mobility of the raw materials in the environment (free water) and the protocells’ potential capability to move around actively. Finally, the permeability of the membrane to raw materials was shown to be a major problem regarding the disadvantage for the cellular form.

**Conclusions:**

The transition from the molecular level to the cellular level may have occurred naturally in early history of evolution. The evolutionary mechanisms for this process were complex. Besides the membrane boundary’s roles to guarantee the molecular cooperation and to resist parasites, the greater chance for the protocells to access raw materials – either due to the diffusion of raw materials outside or the protocells’ active movement, should also be highlighted, which may have at least to an extent compensated the disadvantage regarding the membrane’s blocking effect against raw materials. The present study represents an effort of systematical exploration on this significant transition during the arising of life.

## Background

The emergence of life on our planet is attractive to scientists because it represents one of the most amazing phenomena in nature. However, related experimental researches turn out to be difficult. Indeed, people’s ambitious intention to demonstrate similar processes in laboratory is still far from success – endeavors along this line were initially marked by the famous Miller-Urey experiment in 1950s [1]. A major difficulty comes from the fact that the origin of life is not only a problem of chemistry, but also a problem of evolution [2]. That is, any evolutionary “fragment” involved in the process may have lasted a long time in history (we actually have no idea on the corresponding time span, perhaps years, hundreds of years, or even much longer), and is difficult to mimic or study in the lab. Thanks to the development of computer technology (particularly the improvement of computational capability), it is now becoming more and more effective to tackle the evolutionary aspect through in silico simulation [3–5]. Expectably, the combination of the experimental and the theoretical efforts may greatly promote our understanding on the origin of life, the fascinating problem.

If we do not consider the so-called “chemical evolution”, the evolutionary aspect of the origin of life is just about the preliminary stage of Darwinian evolution. Indeed, Darwinian evolution is an essential feature of the life phenomenon [6–8], and thus its beginning stage constitutes a significant target for the research of the origin of life. In fact, there has already been quite a lot of theoretical work in this area. For example, it has long been suggested that the earliest Darwinian evolution should have run at the molecular level, manifested by molecules capable of replicating (thus, named “replicators”) [9–11]. Then, there should be a transition to the stage of protocells, which have a membrane boundary to hold the replicating molecules together (becoming “reproducers”) [3, 11]. This transition has been referred to as “the first major transition leading to life” [11, 12]. These theoretical studies, with the aid of computer simulation, contributed a lot to our understanding on the earliest Darwinian evolution. However, they generally based on quite abstract models, which limit their relevance to concrete scenarios regarding the origin of life.

The idea of the RNA world hypothesis [13], which has gained a lot of evidence and become popular in the field of the origin of life [14–17], was original raised to solve an “egg-chicken” paradox: if both DNA and proteins are indispensable for life, then which came first in the process of evolution? That is, RNA came first, playing both the roles of DNA (genetic material) and proteins (functional material), and was substituted later in the evolution (because DNA is more stable and proteins are more chemically active) [13, 14]. However, the mentioned paradox is ultimately concerning the starting of Darwinian evolution because, as we know, genetic feature and functional feature are just the two fundamental requirements for Darwinian evolution. In other words, the idea of the RNA world actually provides a solution for the proceeding of Darwinian evolution based upon only one sort of material, thus rendering the starting of Darwinian evolution much “easier” (see [18] for a detailed discussion). However, whether this “world” really stood at the start point of life’s Darwinian evolution is often doubted owing to our ignorance about the prebiotic chemistry which might have launched the RNA world [14, 19]. The relevant difficulties once appeared so great that they were summarized as “the prebiotic chemist’s nightmare” [19]; and such that, alternative scenarios adopting RNA-like polymers (also acting as both genetic and functional material) have been suggested, as “pre-RNA worlds” [19–21].

In fact, the RNA world provides a concrete scenario concerning the origin of Darwinian evolution, and is a best target for theoretic work in this area [18]. Even if a pre-RNA world might turn out to have stood at the start point instead, fundamental mechanisms (in line with “one material dual roles”) remain the same, and the results/conclusions for the RNA world would be easy to “transplant” to this RNA-like world. Therefore, the suspense regarding the de novo origin of the RNA world should not prevent us from modeling this scenario, which aims at investigating detailed mechanisms involved in the earliest Darwinian evolution. On the other side of the coin, it is worth noting that the de novo origin of the RNA world has recently gained strong experimental supports, owing to the reveal of the “new route” of nucleotide synthesis which is “prebiotic plausible” [22–24]. Indeed, as we have seen, now theoretic researchers in this area are increasingly associating their fundamental ideas with the concrete scenario of the RNA world [4, 5].

In the scenario of the de novo origin of the RNA world, it was popularly assumed that a functional RNA that can catalyze its own replication (by template-directed RNA synthesis) – may have emerged first from a prebiotic nucleotide pool [14, 19–21]. There have been long-standing efforts to construct such an RNA “replicase” by in vitro molecular evolution [25–28]. Recently, it was reported that an RNA polymerase ribozyme had been gained capable of copying an RNA template even longer than itself (about 200 nt) [29]. Admittedly, the copying is still limited to special templates – that is, the ribozyme is yet not able to catalyze its own copying; and in addition, a ribozyme about 200 nt seems too long to be able to appear de novo. Alternatively, a ribozyme catalyzes the synthesis of the building blocks of RNA, i.e., nucleotide synthetase ribozyme [30], may have emerged first in the RNA world because it may also favor its own replication [31]. This idea deserves serious consideration especially when considering that it has now been shown that RNA may undergo non-enzymatic template-directed synthesis rather efficiently [32–34] (that is, the RNA replicase is not absolutely necessary for the replication of other RNA species [35]).

No matter how, these two functional ribozymes, which favors the replication of RNA from different aspects, may have both emerged in the “prebiotic pool”, and cooperated with each other. This possibility was first explored by our group [36] and then analyzed in details by Higgs’ group [37] (both by computer simulation, but based upon different models). In the Higgs and coworkers’ study, it was shown that the coexistence of the two ribozymes, though may be evident, is sensitive to quite a few factors. They concluded that “a system of many types of ribozymes would be difficult in a purely spatial model” and then “encapsulation of strands in protocells would have been important fairly early in the history of life”. The conclusion is generally in agreement with our experience and results in previous studies – this is perhaps a little surprising when considering that the type and details of our model are quite different from theirs, which implies the robustness of the conclusion. It seems that before further evolution (i.e., the emergence of more ribozymes), protocells should “show up”.

Therefore, an interesting event – in the next step – is about the emergence of protocells containing the two ribozymes from the naked scene with the two ribozymes. As mentioned above, the transformation from the molecular form to the cellular form was suggested to have been the first major evolutionary transition in life’s history [11, 12]. Obviously, detailed exploration of such a corresponding event in context of the RNA world would provide us an opportunity to check into the evolutionary dynamics of this “revolutionary transformation”.

## Model

The present model is of the same type as those used in our previous studies on the scenario of the RNA world [31, 36, 38–43]. Here we introduce the model in a concise way first, and then explain the details of its implementation in “Methods” at the end of the paper.

### For the naked scene

We assume a two-dimensional system, with an N × N square grid (with toroidal topology to avoid edge effects), in which molecular objects are distributed, including the raw materials to synthesize nucleotides (namely nucleotide precursors, in quotient of nucleotides), nucleotides, and RNAs. Only molecules within the same “grid room” are possible to interact with each other within one time step.

A nucleotide precursor may transform to a nucleotide (randomly as A, U, C, or G), and a nucleotide may also decay into a nucleotide precursor. Nucleotides may join to each other, forming RNAs. Phosphodiester bonds within an RNA chain may break. A nucleotide residue at the end of an RNA chain may decay into a nucleotide precursor. An RNA may act as a template and attract substrates (nucleotides or oligomers) by base-pairing. Substrates aligned adjacently on the RNA template may be ligated to each other. The substrates (or the products) and the template may detach from each other if the base pairs between them separate. A molecule may move to an adjacent (upper, lower, left, or right) grid room. Notably, in the simulation, because each event in the model system occurs with a certain probability within one time step, such a step is also called a “Monte Carlo step” (all entities updates for their relevant events in a Monte Carlo step).

An RNA containing a characteristic catalytic domain (presumed arbitrarily) may function as a ribozyme. The RNA replicase (REP) and the nucleotide synthetase ribozyme (NSR) have different catalytic domain sequences. A short specific sequence (also arbitrarily presumed) at 5′ end of an RNA is assumed to be a tag. REP may bind onto a template by recognizing the tag sequence, and act as a template-directed polymerase (that is, when REP is at working, only mononucleotides – not oligonucleotides – are able to be incorporated successively). In fact, a REP recognizes the tag sequence by base-pairing, and thus, in addition to its catalytic domain, it should also contain a tag-recognizing domain, which is complementary to the tag sequence (see Methods for details).

### For the scene involving protocells

Based on the model for the naked scene, three new kinds of objects, amphiphiles (i.e., membrane components), the raw materials to synthesize the amphiphiles (namely amphiphile precursors, in quotient of amphiphiles) and protocells, are introduced into the system. Each protocell occupies a single grid room.

**Fig. 1:**
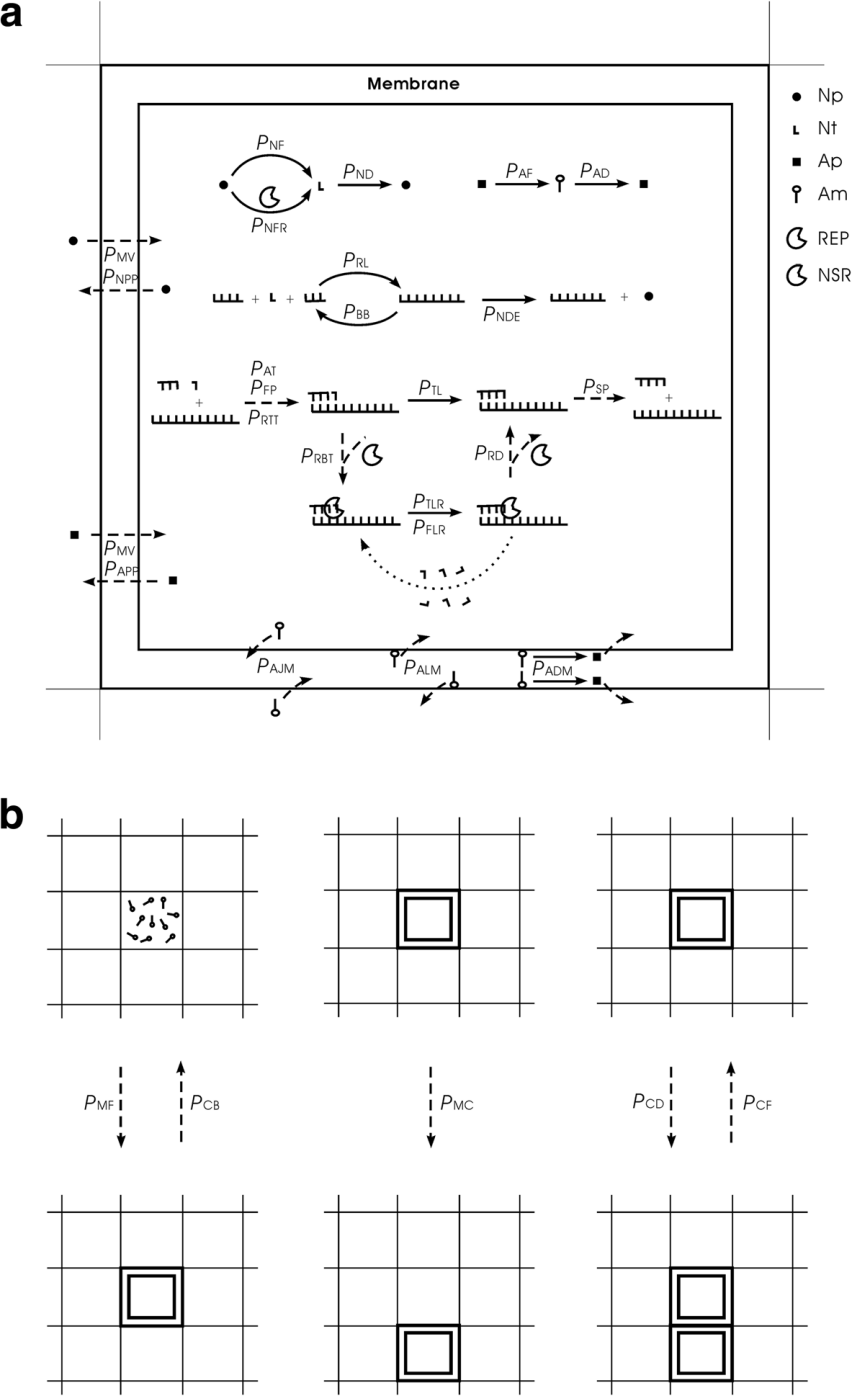
Events occurring in the model for the scene involving protocells. Solid arrows denote chemical reactions and dashed arrows represent other events. The dotted arrow represents the repeating steps involved in the template-directed copying catalyzed by REP. Legends: (Np – nucleotide precursor); (Nt – nucleotide); (Ap – amphiphile precursor); (Am – amphiphile). The events occurring within a protocell are shown in (a). The events concerning the behaviors of the protocells are depicted in (b), which adopts a smaller scale. The event of protocell’s cytophagy (engulfing all molecules of an adjacent room), which is somewhat difficult to depict, is not included here. The model for the naked scene is just a simplified version of this model: no membrane at the edge of the grid room; excluding the events concerning amphiphiles, amphiphile precursors and protocells. See Table 1 for interpretation of the probabilities’ names

An amphiphile precursor may transform to an amphiphile, and an amphiphile may decay into an amphiphile precursor. Amphiphiles (with a lower limit of quantity) may assemble into membrane at the edge of a grid room, encompassing molecules within it and forming a “protocell”. A protocell may also break, with its membrane components turning into free amphiphiles. A free amphiphile may join into a membrane, and an amphiphile on a membrane may leave as well. An amphiphile on a membrane may also decay into an amphiphile precursor, which then leaves the membrane. Nucleotides and RNAs are assumed to be impermeable, whereas nucleotide precursors and amphiphile precursors may diffuse across the membrane. A protocell may engulf the substance (all molecules) in an adjacent room. A protocell may divide into two, and two adjacent protocells may also fuse into one. A protocell may move to an adjacent room. When a protocell divide or move towards an adjacent room, the molecules within the latter are pushed away. Figure 1 is a scheme describing this model. The model for the naked scene (mentioned above) is a simplified version of this one.

## Results

### From the spread of a REP to the co-spread of the REP and an NSR

Here, it should be mentioned that we do not insist either REP or NSR should have emerged first in the naked scene, and the focus of our study is the emergence of protocell from the naked scene with the two ribozymes. However, choosing a route in which one ribozyme emerged and the other followed is helpful for us to envision a complete story about the origin of the RNA world. Additionally, modeling from the very beginning will provide clues for our later analysis on the emergence of protocells, e.g., about the movement of molecules, which is an important factor associated with the effect of membrane boundary (as we will see in the following). To this end, here we followed the classic scenario: “the REP first”, the same as adopted in the work of Higgs’ group [37]. Note that to be more comparable to their work, we adopted a polymerase-typed REP here, instead of a ligase-typed one, which had been adopted in our previous studies [36, 40]. Indeed, in a recent work, using a model of the same type as used here, we have shown that a polymerase-typed REP can spread (becoming thriving through replication – overcoming the parasite problem) in the naked system provided with the so-called “tag mechanism” [43].

**Fig. 2:**
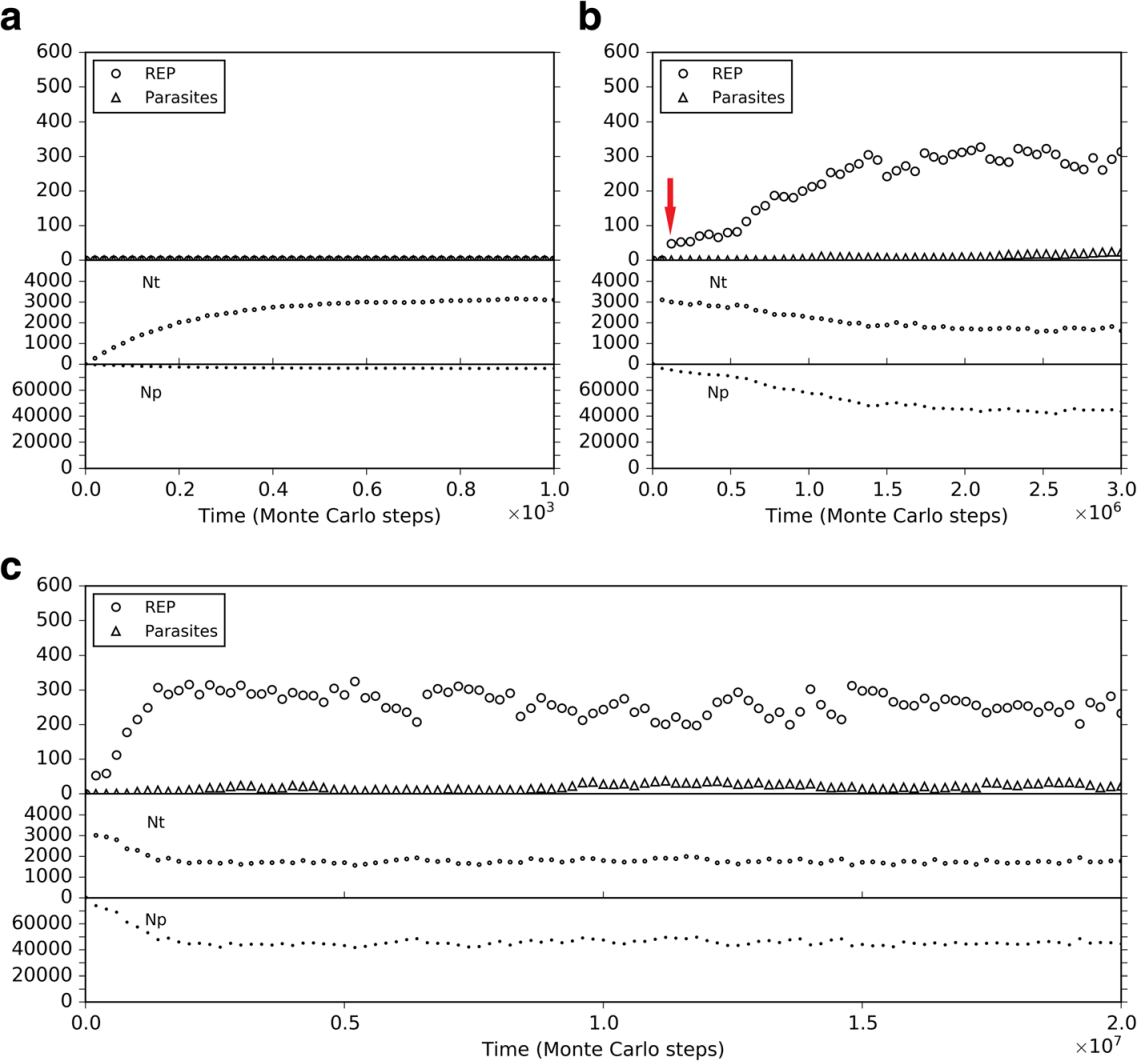
The spread of REP. The vertical axis represents the number of molecules. The system is a 20 × 20 grid (i.e., *N* = 20), 80,000 nucleotide precursors (Np) are introduced in the beginning (i.e. *T*_NPB_ = 80,000), no amphiphile precursors are introduced (i.e. *T*_APB_ = 0), and other parameters adopt default values (Table 1). The subfigures are showing the same case with different time scales. **a** A balance between nucleotides and their precursors is reached soon. **b** 50 REP molecules with a sequence of “*CS*_Tag_ + *CS*_REP_ + *CS*_Tag_” (see Methods for explanation, Fig. 16) are inoculated at step 1 × 10^5^ (see arrow) into the system (distributed in 10 adjacent grid rooms at center, five each). The REP spreads in the system and reaches a balance. Parasites (in the context of this paper) mean the RNA molecules with two end-*CS*_Tag_s, but without a *CS*_REP_ (i.e., with another sequence instead, or no corresponding sequence) in between. **c** The REP’s balance lasts, but more than a half of the nucleotide precursors remain unused

Nucleotide precursors of a certain quantity (*T*_NPB_; see Table 1 for the descriptions of parameters) are introduced in the very beginning. They may transform to nucleotides (with probability *P*_NF_), and the nucleotides, in turn, may decay into nucleotide precursors (with *P*_ND_). A balance between nucleotides and their precursors may be reached soon (Fig. 2a). The balance ratio is determined by the relative scale of *P*_NF_ and *P*_ND_. According to the model, nucleotides may join with each other end-to-end (with *P*_RL_) and form RNAs. One REP molecule may appear this way by chance; and then by non-enzymatic template-directed copying, derive its complementary chain. The separation of the two chains would give rise to a ribozyme and a template (the complementary chain), thus launching the REP-catalyzed template-directed replication. Theoretically, the template (the REP’s complement or a second REP) molecule may also appear by random ligation, though less likely. No matter how, such de novo emergence of ribozymes has been demonstrated in our previous modeling studies to be feasible [31, 40]. Here, to focus on our current topic, we omit such a modeling process, which is arduous. Instead, we “inoculate” a certain number of REP molecules into our system (Fig. 2b, see arrow) to observe whether they could spread. The outcome of the simulation is in agreement with the one in our previous study [43] on the tag-mediated polymerase-typed REP (though the details of the tag mechanism adopted here is a little different, see Methods).

**Table 1:** Parameters used in the computer simulation

	Descriptions	Magnitudes ^a^	Default Values ^b^
Probabilities			
ᅟ*P*_AT_	An RNA template attracting a substrate (by base-pairing)	[0.3, 0.5]	0.4
ᅟ*P*_BB_	A phosphodiester bond breaking within an RNA chain	[1 × 10^− 6^, 5 × 10^− 6^]	2 × 10^− 6^
ᅟ*P*_FLR_	The false ligation under the catalysis of a REP	[0.05, 0.2]	0.1
ᅟ*P*_FP_	The false base-pairing when a template attracts a substrate	[5 × 10^−5^, 0.0002]	0.0001
ᅟ*P*_MV_	The movement of nucleotides/amphiphiles or their precursors	[5 × 10^−5^, 0.0002]	0.0001
ᅟ*P*_ND_	A nucleotide decaying into its precursor	[0.002, 0.01]	0.005
ᅟ*P*_NDE_	A nucleotide residue decaying at RNA’s chain end	[0.0001, 0.0005]	0.0002
ᅟ*P*_NF_	A nucleotide forming from its precursor (non-enzymatic)	[0.0001, 0.0005]	0.0002
ᅟ*P*_NFR_	A nucleotide forming from its precursor catalyzed by an NSR	[0.5, 0.9]	0.9
ᅟ*P*_RBT_	A REP binding onto the template containing a tag	[0.3, 0.5]	0.4
ᅟ*P*_RD_	A REP dropping from a template	[5 × 10^−5^, 0.0002]	0.0001
ᅟ*P*_RL_	The random ligation of two RNAs (including nucleotides)	[5 × 10^−7^, 2 × 10^−6^]	1 × 10^− 6^
ᅟ*P*_RTT_	An RNA turning to a template (for non-enzymatic synthesis)	[0.4, 0.6]	0.5
ᅟ*P*_SP_	The separation of a base pair	[0.3, 0.4]	0.38
ᅟ*P*_TL_	The template-directed ligation (non-enzymatic)	[5 × 10^−5^, 0.0002]	0.0001
ᅟ*P*_TLR_	The template-directed ligation catalyzed by a REP	[0.5, 0.9]	0.9
ᅟ*P*_AD_	An amphiphile decaying (out of membrane)	[0.0001, 0.0005]	0.0002
ᅟ*P*_ADM_	An amphiphile decaying within membrane	[1 × 10^−5^, 5 × 10^−5^]	2 × 10^−5^
ᅟ*P*_AF_	An amphiphile forming from its precursor	[0.0005, 0.002]	0.001
ᅟ*P*_AJM_	An amphiphile joining the membrane	[0.7, 0.9]	0.8
ᅟ*P*_ALM_	An amphiphile leaving the membrane	[0.0001, 0.0005]	0.0002
ᅟ*P*_APP_	An amphiphile precursor permeating the membrane	[0.5, 1]	1
ᅟ*P*_CB_	A protocell breaking	[5 × 10^−6^, 2 × 10^−5^]	1 × 10^−5^
ᅟ*P*_CC_	A protocell engulfing molecules (cytophagy)	[1 × 10^−7^, 5 × 10^−7^]	1 × 10^−7^
ᅟ*P*_CD_	A protocell dividing	[5 × 10^−5^, 0.0002]	0.0001
ᅟ*P*_CF_	Two adjacent protocells fusing with each other	[0.002, 0.01]	0.005
ᅟ*P*_MC_	The movement of a protocell	[5 × 10^−6^, 2 × 10^−5^]	1 × 10^− 5^
ᅟ*P*_MF_	A membrane forming	[0.005, 0.02]	0.01
ᅟ*P*_NPP_	A nucleotide precursor permeating the membrane	[0.05, 0.2]	0.1
Others			
ᅟ*N*	The system is defined as an N × N grid	[20, 40]	40
ᅟ*T*_NPB_	Total nucleotide precursors introduced in the beginning	[80,000, 320,000]	320,000
ᅟ*T*_APB_	Total amphiphile precursors introduced in the beginning	[60,000, 240,000]	240,000
ᅟ*F*_OP_	The factor for the effect of osmotic pressure	[5, 10]	10
ᅟ*F*_DE_	The factor for the effect of Donnan’s equilibrium	[5, 10]	10
ᅟ*C*_T_	Collision times in a step	[5, 10]	8
ᅟ*L*_AM_	The lower limit number of amphiphiles to form a membrane	[400, 600]	500
ᅟ*CS*_REP_	The characteristic (catalytic domain) sequence of REP	8–10 nt long	CUCGACAGAU
ᅟ*CS*_NSR_	The characteristic (catalytic domain) sequence of NSR	8–10 nt long	ACUGGCAUCU
ᅟ*CS*_Tag_	The characteristic sequence of the tag	3–5 nt long	ACGU

Note: The upper half of the probabilities include those for the naked scene (with names in alphabetical order), whereas the lower half are those introduced when the scene involving protocells is modeled (with names in alphabetical order). **a.** The magnitudes represent the general scopes of the values that were adopted in our study, but in the cases for investigating some parameters specially (see text), a broader range may be explored; and in some special cases, some key parameters may also adopt values beyond this scope (see text). **b.** The simulation cases shown in this paper adopt the default values listed here, unless being stated explicitly to be different

**Fig. 3:**
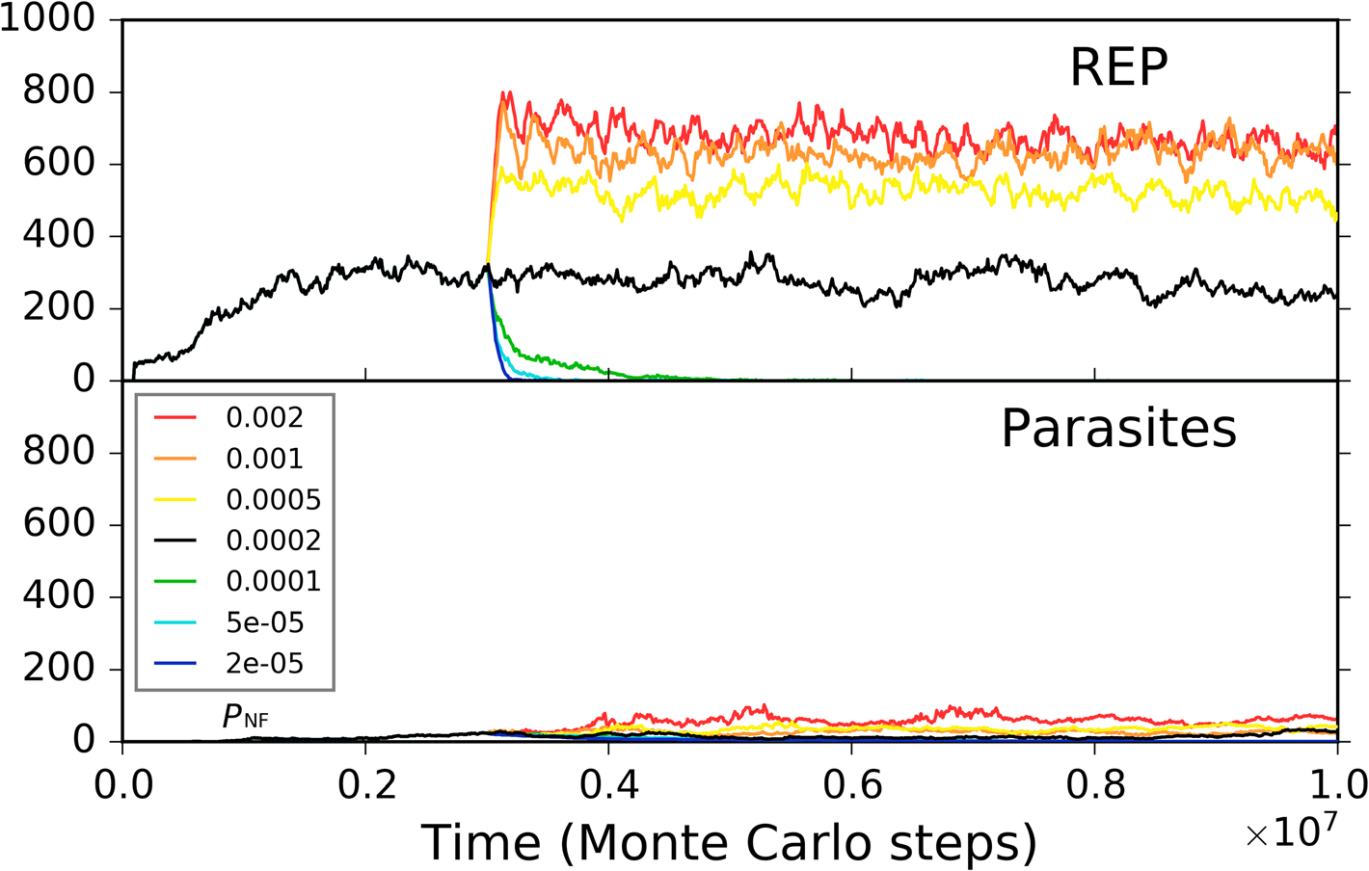
The influence of non-enzymatic nucleotide-synthesis rate on the spread of REP. The case shown in black is the same as the one of Fig. 2. At step 3 × 10^6^, *P*_NF_ is changed, deriving the cases shown in other colors. The color legends at the lower left apply to both of the two subfigures. Notably, here we use different *P*_NF_ values until REP reaches a stable level instead of from the beginning. If we adopt a low *P*_NF_ from the beginning (the green, cyan, and blue lines), we would only see that REP does not rise and spread, and will never see the more impressive result that a low *P*_NF_ may even “destroy” a REP-thriving system; and we would not observe that the influence of different low *P*_NF_ values may be different – the lowest *P*_NF_ would be the worst (the blue line). In fact, changing the values in the midway of one case may demonstrate more directly and vividly the influence of the parameter on the dynamics of the model system. Similar consideration has also been taken in some other analyses shown below

During the spreading of the REP, we see that both nucleotides and their precursors shift towards a lower level (Fig. 2b). This is not surprising, because they are exploited by the REP for its replication – directly the nucleotides, and then their precursors due to the *P*_NF_ event. However, there could still be a lot of the raw materials, e.g., here more than a half of the initial nucleotide precursors remain unused even after the REP reaches its “thriving balance” (Fig. 2b, and also Fig. 2c for the long-run situation). In fact, this is owing to our assumption of a low *P*_NF_ value. Figure 3 shows several different cases which derive from the case of Fig. 2 by changing the *P*_NF_ value at a middle step. Indeed, a higher *P*_NF_ would obviously favor the thriving of the REP, which should be attributed to a higher rate of exploiting the raw materials in the system. However, a prebiotic route to produce nucleotides is not likely to have been quite efficient. Thus, there should have been an obvious selective pressure for the emergence of a ribozyme catalyzing the formation of nucleotides from their precursors, i.e., NSR.

**Fig. 4:**
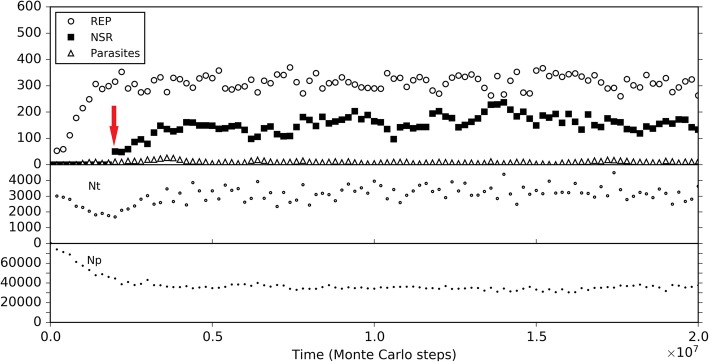
The spread of NSR in the system with REP thriving. Based upon the case of Figs. 2, 50 NSR molecules with a sequence of “*CS*_Tag_ + *CS*_NSR_ + *CS*_Tag_” (see Methods for explanation) are inoculated at step 2 × 10^6^ (see arrow) into the system (distributed in 10 randomly chosen grid rooms containing REP, five each)

**Fig. 5:**
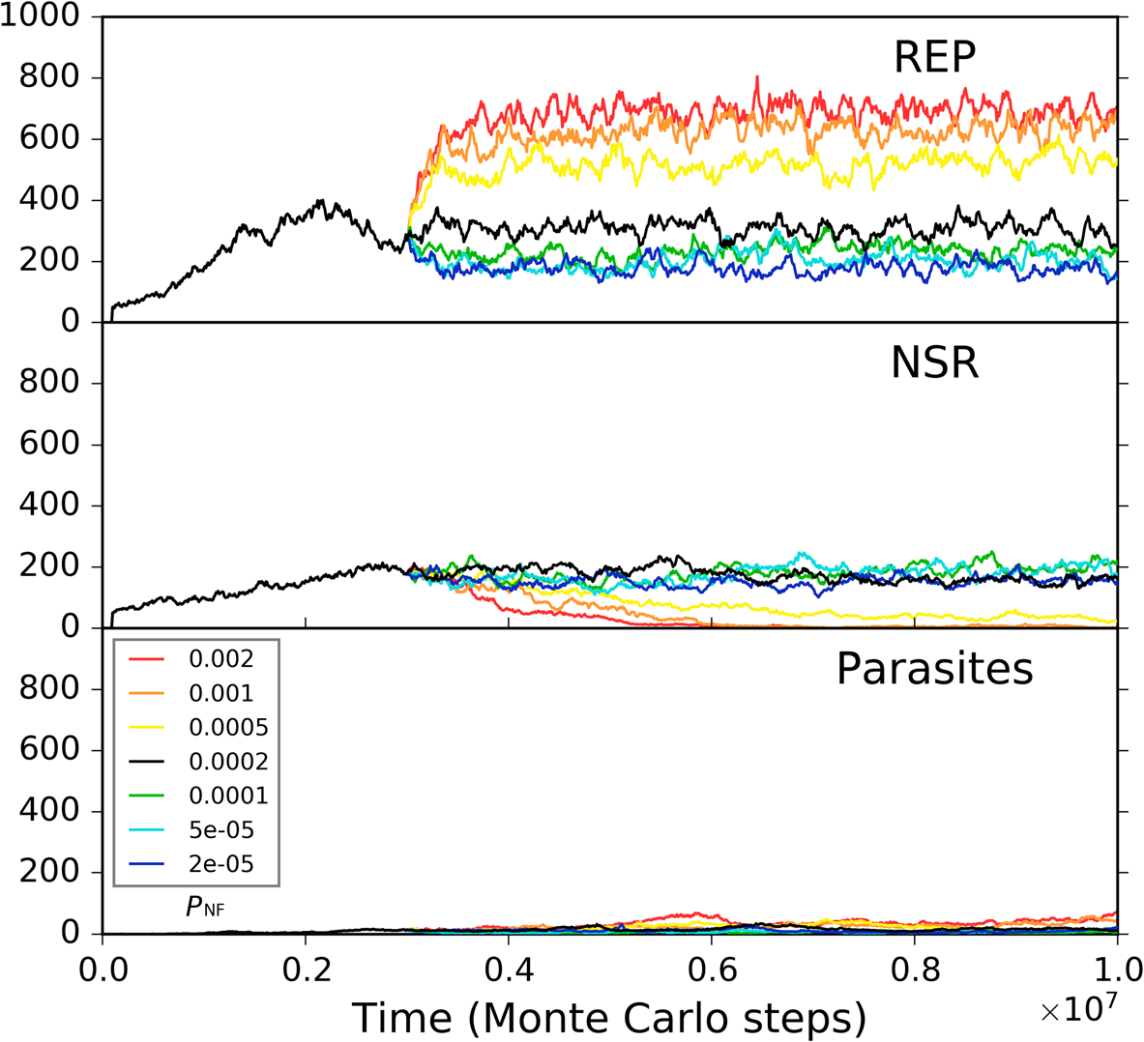
The influence of non-enzymatic nucleotide-synthesis rate on the co-spread of REP and NSR. The parameter values are identity to those adopted in the case of Fig. 2. 50 REP molecules with a sequence of “*CS*_Tag_ + *CS*_REP_ + *CS*_Tag_” and 50 NSR molecules with a sequence of “*CS*_Tag_ + *CS*_NSR_ + *CS*_Tag_” are inoculated at step 1 × 10^5^ into the system (distributed in 10 adjacent grid rooms at center, five REP molecules and five NSR molecules for each room). The two ribozymes co-spread in the system – the case shown in black. At step 3 × 10^6^, *P*_NF_ is changed, deriving the cases shown in other colors. The color legends at the lower left apply to all the subfigures

So we inoculate a certain number of NSR to observe whether it can spread in the REP-thriving system (Fig. 4). The result is consistent with our expectation – the NSR co-spreads with the REP in the system. In comparison with the situation before the inoculation of NSR, nucleotides increase and nucleotide precursors decrease, because the NSR functions to synthesize nucleotides from their precursors. Generally, the fluctuation (increasing/decreasing) of nucleotides is synchronous as the fluctuation of NSR. Additionally, we can observe a clear reverse-synchronicity between the REP and NSR – that is, when the REP increases, the NSR decreases, and vice versa. This is a clear indication of the competition between the two RNA species, which is easy to appreciate, given that both of them exploit the same building blocks in their replication. On the other hand, notably, such a type of oscillation is also an indication of cooperation between the two ribozymes – when the REP rises to a certain high level (relative to its own oscillation) and the NSR descends to a certain low level (relative to its own oscillation), why this tendency does not continue? The reason is that the bottle-neck reaction would then become the nucleotide synthesis, and the NSR would be more favored – it would rise back, supplying nucleotides for both of the RNA species. Similarly, when the NSR rises to a high level and the REP descends to a low level, the bottle-neck reaction would become the template-directed synthesis of RNA – the REP would rise back, catalyzing the template-directed copying for both of them. Indeed, we can see the sign of cooperation more than competition from another angle – even when NSR, as a new RNA species, spreads in the system, the balance level of the REP generally does not descend – instead, the raw materials are exploited towards a greater extent (this is an evolution towards a more thriving living world). To show the cooperation more clearly, we investigate the influence of *P*_NF_ to the co-spread system (Fig. 5). In accordance with the results above (Fig. 3), a higher *P*_NF_ benefits the thriving of REP. However, NSR is obviously disfavored when *P*_NF_ becomes sufficient high, because its function may become dispensable – the cooperation would no longer be so important and the REP may “fly solo”.

**Fig. 6:**
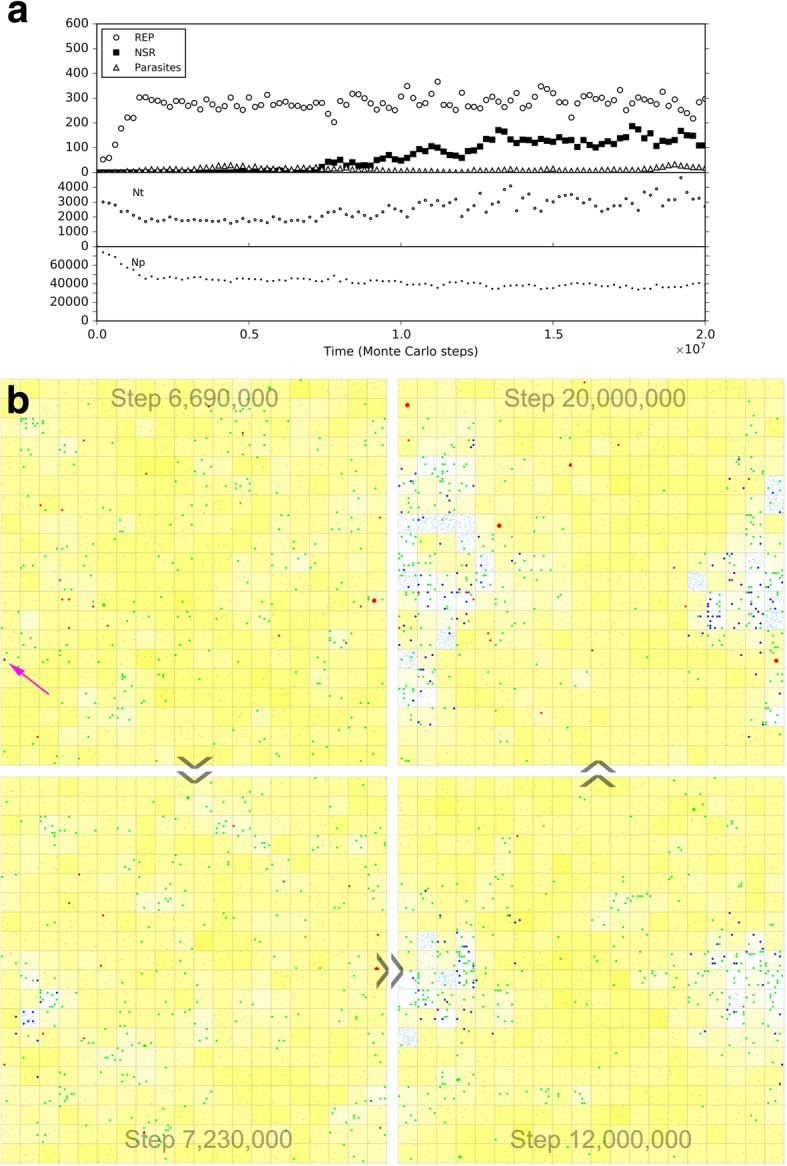
The natural emergence of NSR in a REP-thriving system. This case adopts the same parameter values as the case of Fig. 2, but with a different random seed. It is assumed that *CS*_NSR_ is “CUCGAGAGAU”, only one base different from *CS*_REP_, which is “CUCGACAGAU”. **a** The dynamic plot. **b** Snapshots showing the appearance of the first NSR molecule (arrow) and the subsequent spread of NSR in the system (note that the grid has a toroidal topology, i.e., the left edge is actually adjacent to the right edge and the upper edge is actually adjacent to the lower edge). Raw materials (nucleotide precursors) are shown as yellow background, with color depth representing their quantity in the corresponding grid room. Nucleotides are denoted by tiny cyan dots (if you are reading the e-version you may zoom in for a closer observation). Solid circles denotes REP (green), NSR (blue) and parasites (red), with diameters in scale of molecular sizes (RNA chain lengths)

Having known that NSR might be able to spread in a REP-thriving scene, it would then be attractive to demonstrate a corresponding natural evolution involving the de novo appearance of NSR in the scene. However, the efforts to establish de novo events are usually arduous (as mentioned above concerning the de novo appearance of the REP). Indeed, the thriving of REP would bring out a lot of RNA chains, shorter or longer, with different sequences, which derive from REP’s mutation, degradation, partially replication – and subsequent random ligation or recombination; these would provide a better chance for the NSR to appear de novo in this scene than its de novo appearance from a nucleotide pool. Nonetheless, we found that the exploration of the de novo appearance of NSR involving such random events remain difficult, and as we can understand, to insist a near-reality situation at this point would make little sense. Therefore, here we assumed that the characteristic sequence of the NSR is just one nucleotide residue different from that of the REP, and thus NSR may derived from REP just by a single point mutation during replication. Then we tested different cases by changing the random seed of our simulation to see if the NSR can appear naturally in a REP-thriving system. Finally, we observed such cases (e.g., Fig. 6a; and see Fig. 6b for the corresponding snapshots of the spatial spreading). One case like this, though not likely to be possible in reality because of the merely one-nucleotide difference between the two ribozymes, does, at least, vividly show the obvious selective pressure mentioned above – for the emergence of NSR after the thriving of REP.

### About the movement of molecules

Before moving on to explore the process concerning the emergence of protocells, it would be valuable to check into the problem of molecular movement in the naked scene – for us to understand the significance of the protocell’s membrane. By theoretical studies, it has long been shown that spatial limitation is crucial for the REP-like replicators to resist parasites in the naked stage [3, 4, 44, 45]. About the actual environments, for example, it was imagined that mineral surfaces [46–49], lacunose ices [50–52], or porous rocks [53–55] may have provided the spatial limitation and become the hatchery of early life on the earth. However, in reality, the spatial limitation should be a “big concept”, which is associated with the movement of the ribozymes, the movement of parasites, and the movement of raw materials, as a whole. In our system, indeed, these different kinds of molecular movement are all bound with a basic probability: *P*_MV_, which is associated with the “big spatial limitation”. While a nucleotide precursors or a nucleotide may move with *P*_MV_, an RNA molecule may move with *P*_MV_/*m*^1/2^, where *m* is the mass of the RNA, relative to a nucleotide (see Methods for a detailed explanation). That is, for example, when the basic probability *P*_MV_ increases, the raw materials (nucleotide precursors), the ribozymes and the parasites, all of them, would “simultaneously” move faster. However, considering that the three kinds of molecular movement would mean different things for a membrane boundary, we decided to analyze them separately here.

**Fig. 7:**
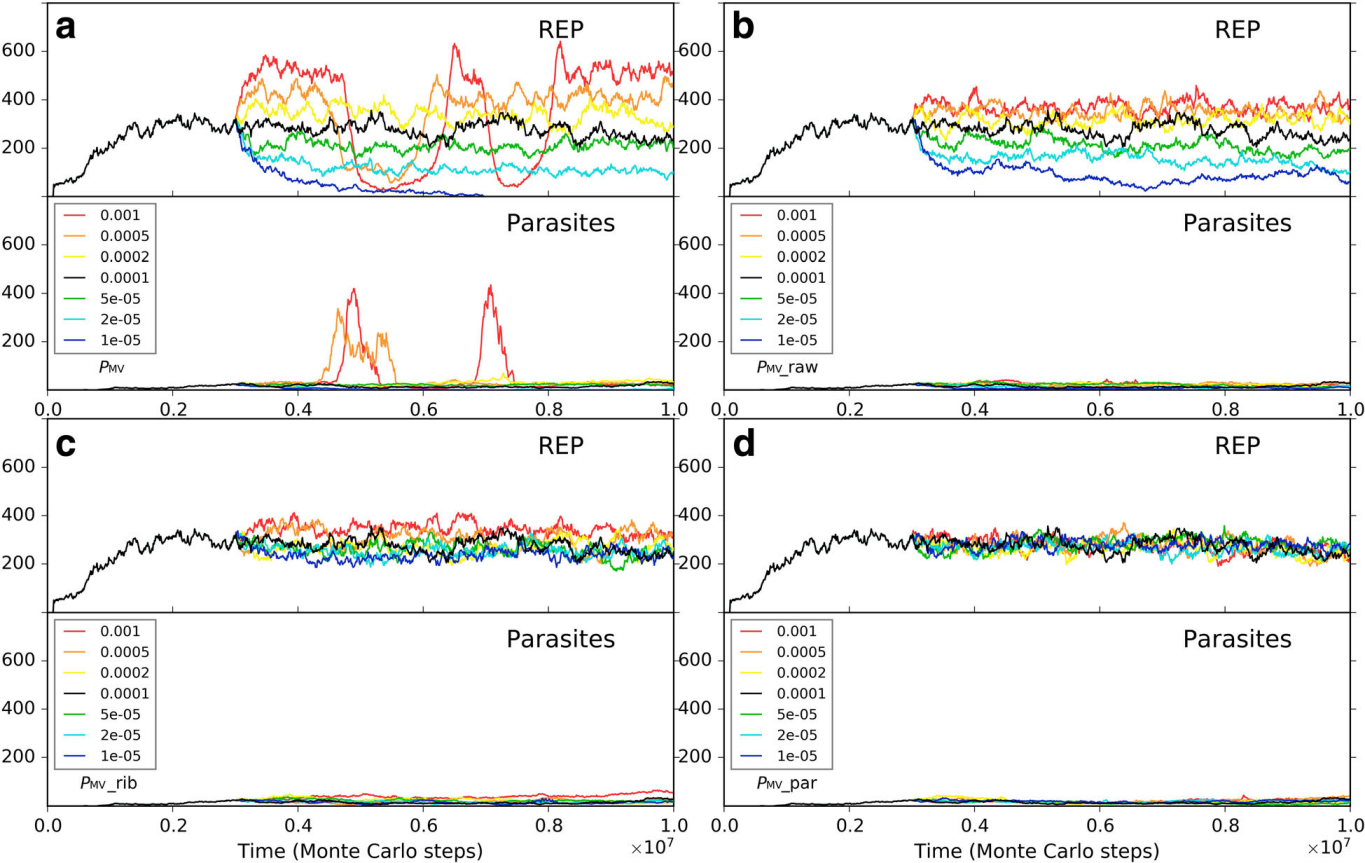
The influence of molecular movement on the spread of REP. The case shown in black is the same as the one of Fig. 2. At step 3 × 10^6^, the moving rate of molecules is changed, deriving the cases shown in other colors. **a** The changed rate is *P*_MV_, which determines the movements of all molecules. Note that the parasites recorded here and those shown in Fig. 8 appear de novo in the system, which have been traced by us and turn out to be the strongest parasites – i.e., the shortest ones able to be replicated continuously, with merely a sequence of “*CS*_Tag_ + *CS*_Tag_”. **b** The changed moving rate is of raw materials (i.e., nucleotide precursors). **c** The changed moving rate is of ribozymes (i.e., REP). **d** The changed moving rate is of parasites (i.e., other RNAs with two end-*CS*_Tag_s)

Firstly, we investigated the situation for REP alone. A low *P*_MV_ (as a whole) disfavors the thriving of the REP, but a *P*_MV_ too high would cause large-scale invasions of parasites (i.e., other RNAs which can also exploit the REP; here they arise de novo in the system), which threatens the stability of the REP’s thriving (Fig. 7a). Such unstability may manifest as a kind of oscillation – after a large-scale invasion of parasites, the REP may recover; but then a second turn of invasion may occur, and the REP descends again (e.g., Fig. 7a, the red line; in fact, similar oscillation can often be observed for predators and preys in an ecosystem). Then, only the change of the movement of raw materials (nucleotide precursors) is allowed (Fig. 7b). Obviously, a higher rate of raw materials’ movement favors REP’s thriving, which should be on account of the greater accessibility of the ribozyme to the raw materials in its replication. When only the movement of the REP is allowed to change, we can find that a faster movement of the ribozyme is also welcome, which should be associated with the issue of accessibility to raw materials as well (Fig. 7c). Combining these two results, that is, it appears that the disadvantage of the low *P*_MV_ (Fig. 7a) could just be ascribed to the less accessibility of the REP to the raw materials in its replication. When only the movement of parasites is allowed to change, somewhat unexpectedly, no obvious tendency is observed (Fig. 7d). In other words, the invasions of parasites at a high *P*_MV_ (Fig. 7a) cannot be attributed to the high dispersal of the parasites themselves, but should be on account of the high dispersal of the whole system. For example, we can imagine that if the ribozyme molecules are limited in space locally, the parasites themselves might be difficult to spread globally, even if they can “wander” freely elsewhere (they cannot find “REP” there).

**Fig. 8:**
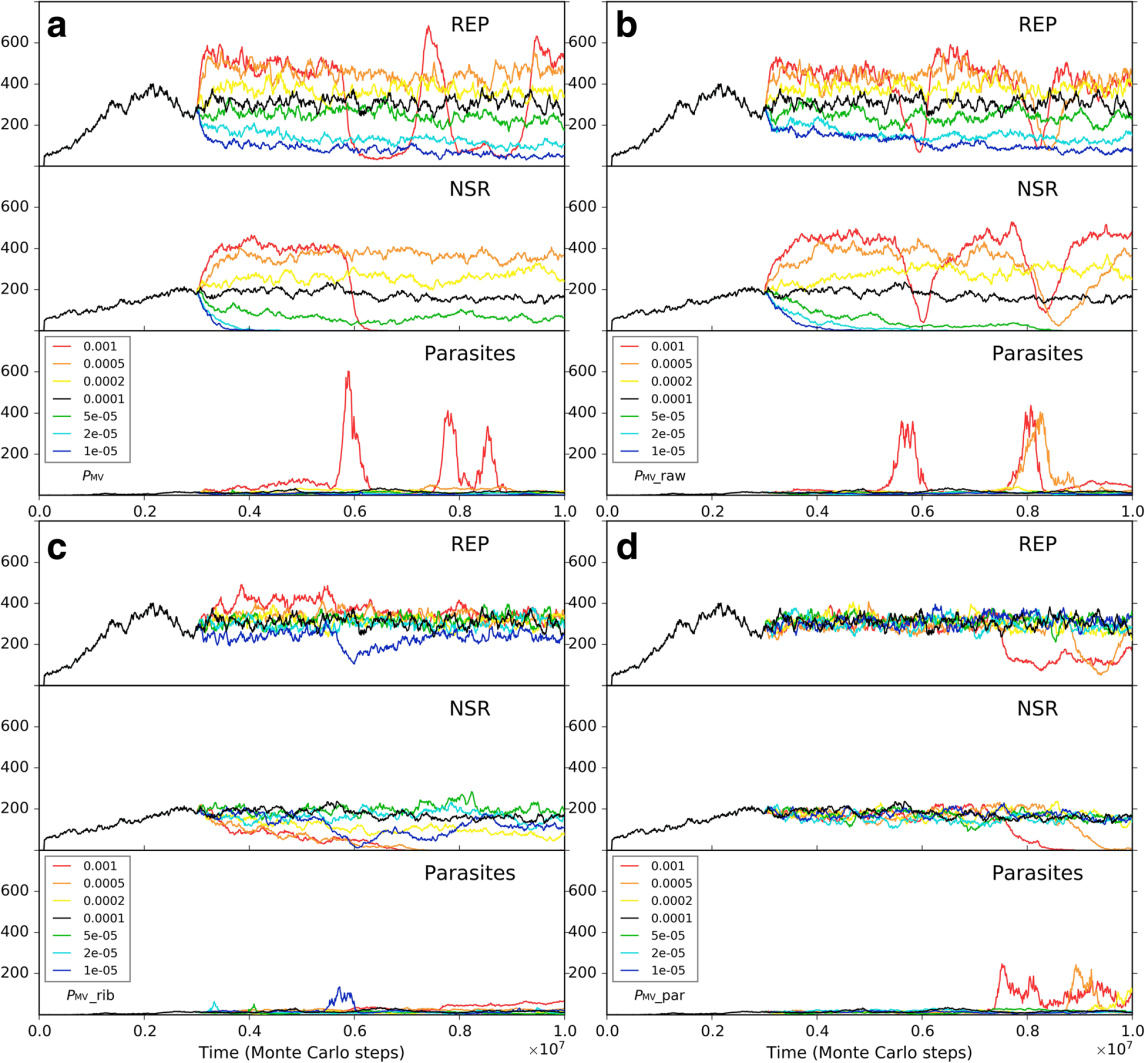
The influence of molecular movement on the co-spread of REP and NSR. The case shown in black is the same as the one of black line in Fig. 5. At step 3 × 10^6^, the moving rate of molecules is changed, deriving the cases shown in other colors. **a** The changed rate is *P*_MV_, which determines the movements of all molecules. **b** The changed moving rate is of raw materials (i.e., nucleotide precursors). **c** The changed moving rate is of ribozymes (i.e., REP and NSR). **d** The changed moving rate is of parasites (i.e., other RNAs with two end-*CS*_Tag_s)

Secondly, we investigated the situation for the co-thriving of REP and NSR. The influence of *P*_MV_, as a whole, is similar to the situation of REP alone. A low *P*_MV_ disfavors the thriving of both REP and NSR, but a *P*_MV_ too high would cause large-scale invasions of parasites, which threatens the stability of the two RNA species’ thriving (Fig. 8a, note that in the red-line case NSR is completely destroyed during a parasite invasion). When only the moving rate of raw materials (nucleotide precursors) is changed, a little unexpectedly, the situation is similar to that of the whole *P*_MV_ (Fig. 8b). That is, the influence of the movement of raw materials is obviously stronger than the situation for REP alone (Fig. 7b) – here, a greater mobility of raw materials would result in a higher level of REP, and may even lead to parasite invasions (we see both the oscillation of REP and NSR caused by the invasions; Fig. 8b, the red and orange lines). The reason should be that the introduction of NSR enhances the effect regarding the accessibility to nucleotide precursors since the function of this ribozyme is just to increase the rate of exploiting nucleotide precursors – to synthesize nucleotides, the building blocks of RNAs. Additionally, we can notice that NSR is more sensitive to the decrease of the raw materials’ mobility than REP (Fig. 8b), which is also on account of the NSR’s function – a less availability of these nucleotide precursors might sharply reduce this ribozyme’s advantage. When only the moving rate of the ribozymes is changed, the effects are a bit complex (Fig. 8c). A faster movement of the ribozymes, generally, favors the REP (but perhaps not so straightforward when compared with the situation of REP alone, see Fig. 7c). However, a greater mobility of ribozymes disfavors the thriving of NSR clearly. This phenomenon should just represent the requirement of proximity in the cooperation of the two ribozymes. If the ribozymes move too fast, it might be difficult for NSR to “catch up with” REP – but the REP, from the very beginning on, can “fly solo” in our system (e.g., related to the assumptions in regard to the parameter setting). Certainly, losing the help of NSR, REP also decrease to a level (see the red line) – just accounting for the point “not so straightforward” mentioned above about the REP’s reaction to a faster mobility of ribozymes. Finally, when only the moving rate of parasites is allowed to change, interestingly, we see that, different from the REP alone case (Fig. 7d), a movement of parasites sufficiently high may cause the collapse of the two-ribozyme system (Fig. 8d, the red and the orange lines). We suppose that with greater mobility parasites may travel between REP-rich areas to NSR-rich areas more readily, thus more likely to exploit both of the ribozymes (whereas the two ribozymes are more difficult to exploit each other on account of the spatial distance). No matter how, this may be read as an indication that two ribozymes’ co-spread is more sensitive to parasites than one ribozyme’s spread alone, especially when considering parasites in reality tend to be smaller in size than the ribozymes – actually with greater mobility.

Taken together, we can make three conclusions: the first, a higher rate of relative movement between raw materials and ribozymes, which results in a higher accessibility of the RNA species to the raw materials they need in their replication, would favor the spread of the ribozymes; the second, in the co-spread of the two ribozymes, their movement relative to each other should be restricted for the sake of their cooperation; the third, the co-spread of cooperative ribozymes may be more sensitive to parasites than the spread of one ribozyme alone. Interestingly, the emergence of membrane as a clear boundary, which encompasses the RNA species within a vesicle – engendering the so-called RNA-based protocells, seems to be able to make improvements in all the three aspects: the membrane limits the relative motion of the cooperative ribozymes within the vesicle while allowing a great relative motion between raw materials and ribozymes (provided that the raw materials can permeate into the vesicle), and in the meantime, serves as a physical barrier against parasites’ invasion from outside. Then, in the following, we will explore the scenario about the emergence of protocells from the REP-NSR’s co-thriving system in a naked scene. We ask: Are the RNA-based protocells indeed able to emerge from the naked scene with the two ribozymes (provided with a near-reality model)? May the protocells substitute the naked ribozymes therein? If so, what are the detailed mechanisms concerning the superiority of the protocells in comparison with the naked scene? As the other side of the coin, are there any significant disadvantages or limitation regarding the advent of the membrane boundary?

### The emergence of protocells containing REP and NSR

To modeling the emergence of protocells, we constructed a concrete scene using our model. We assumed that at the center of a 40 × 40 grid system, there is a 20 × 20 grid region, which represent a naked subsystem, where REP and NSR co-spread. The scale of the naked subsystem was chosen in part to consider the comparability of the results below with those above (note that the cases of the naked scene shown above is run in a 20 × 20 grid system). We can imagine that the central subsystem is one of the porous rocks mentioned in the submarine-hydrothermal-vent hypothesis [53–55]. The sides of our grid rooms here may represent the walls of those “inorganic compartments” [54], which provides a natural mechanism of the spatial limitation needed for the ribozymes’ spread in the naked situation. The grid rooms outside the central rocky region represent the solution region, i.e., a “water body”. Compared with the rocky region, in the solution region, the probability concerning molecular movement (*P*_MV_) should be higher; the rates concerning with degradation, such as *P*_BB_, *P*_ND_, *P*_NDE_, and *P*_AD_, should be higher, owing to the higher chemical activity of free water (this does not apply to the situation within a protocell, which is not a free water environment); and the rates related to synthesis, such as *P*_RL_ and *P*_NF_, should be lower, due to the shortage of (rock) surface-mediated catalysis. With this consideration, we constructed a system of two phases, between which material-exchange is allowed.

**Fig. 9:**
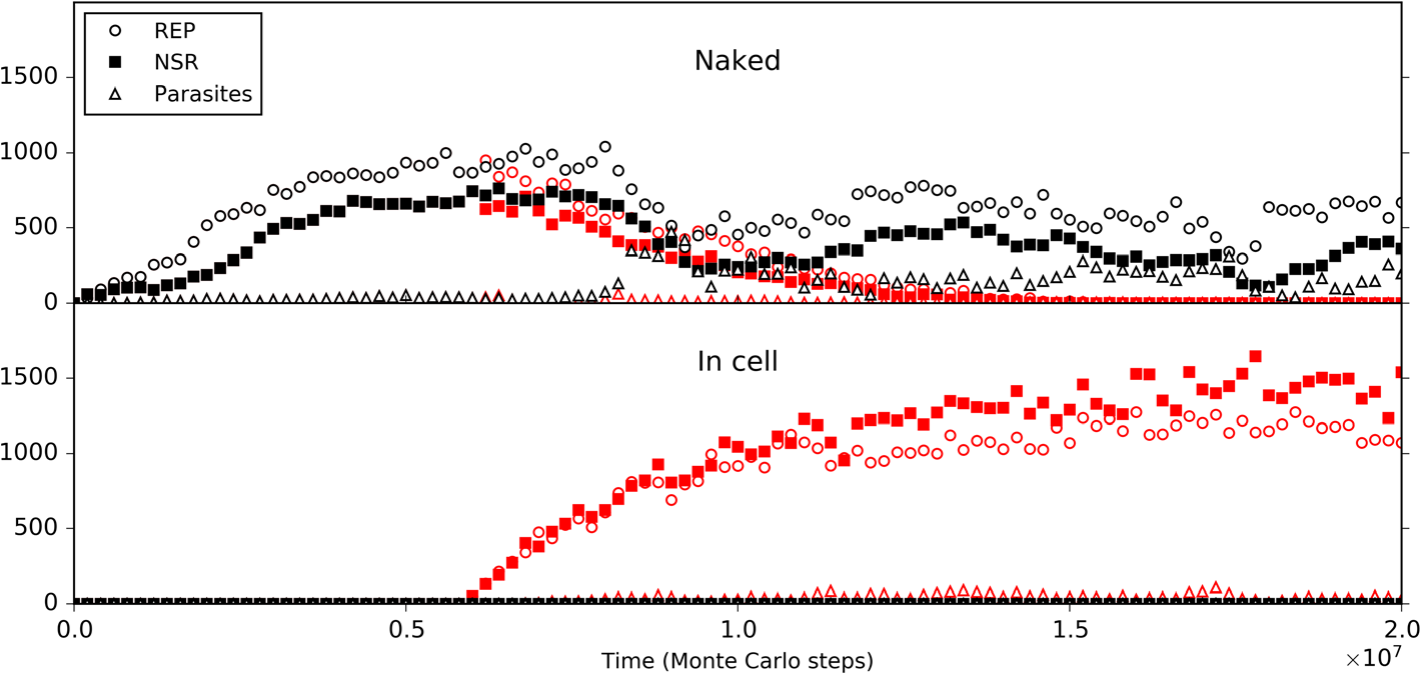
The spread of protocells and the descending of naked ribozymes in a near-reality system. The system is a 40 × 40 grid, which has a 20 × 20 rocky region in the center and a solution region outside the rocky region (see Fig. 10b for a reference). The rocky region corresponds to the naked scene, running with default parameter values list in Table 1. In the solution region, compared with the rocky region, the moving rate of molecules (*P*_MV_) is ten times; the rates associated with degradation(including *P*_BB_, *P*_ND_, *P*_NDE_ and *P*_AD_) are twice (but not apply to the inside of protocells); the rates associated with synthesis (including *P*_RL_ and *P*_NF_) are one twentieth. Similar to the black-line case in Fig. 5, 50 REP molecules and 50 NSR molecules are inoculated at step 1 × 10^5^ into the rocky region. The black symbols in the upper panel demonstrate the evolutionary dynamics of the naked scene without the inoculation of protocells. Based upon this case, at step 6× 10^6^, ten protocells, each containing five REP molecules and five NSR molecules, are inoculated into the solution region (at randomly chosen grid rooms), deriving the case represented by red symbols. The lower panel shows the variation of the number of REP and NSR molecules inside protocells

Initially, we inoculated REP and NSR into the central naked subsystem, and modeled the co-spread of REP and NSR in the rocky region. Similar to the naked scene alone above, REP and NSR co-spread in the subsystem (Fig. 9, black symbols), and here the co-spread is not so stable owing to the occasional invasion of parasites (as we have seen, this is not surprising for a naked scene). Then, we inoculated protocells containing the two ribozymes into the surrounding solution region. Interesting, we found that the ribozymes within the protocells may increase and the protocells spread in the solution region (note: protocells cannot enter the rocky region); meanwhile, the ribozymes in the naked rocky region may decrease and finally become extinct (Fig. 9, red symbols). That is to say, if protocells can appear, they may spread and even substitute the naked ribozymes, implementing the so-called “first major transition” in evolution.

**Fig. 10:**
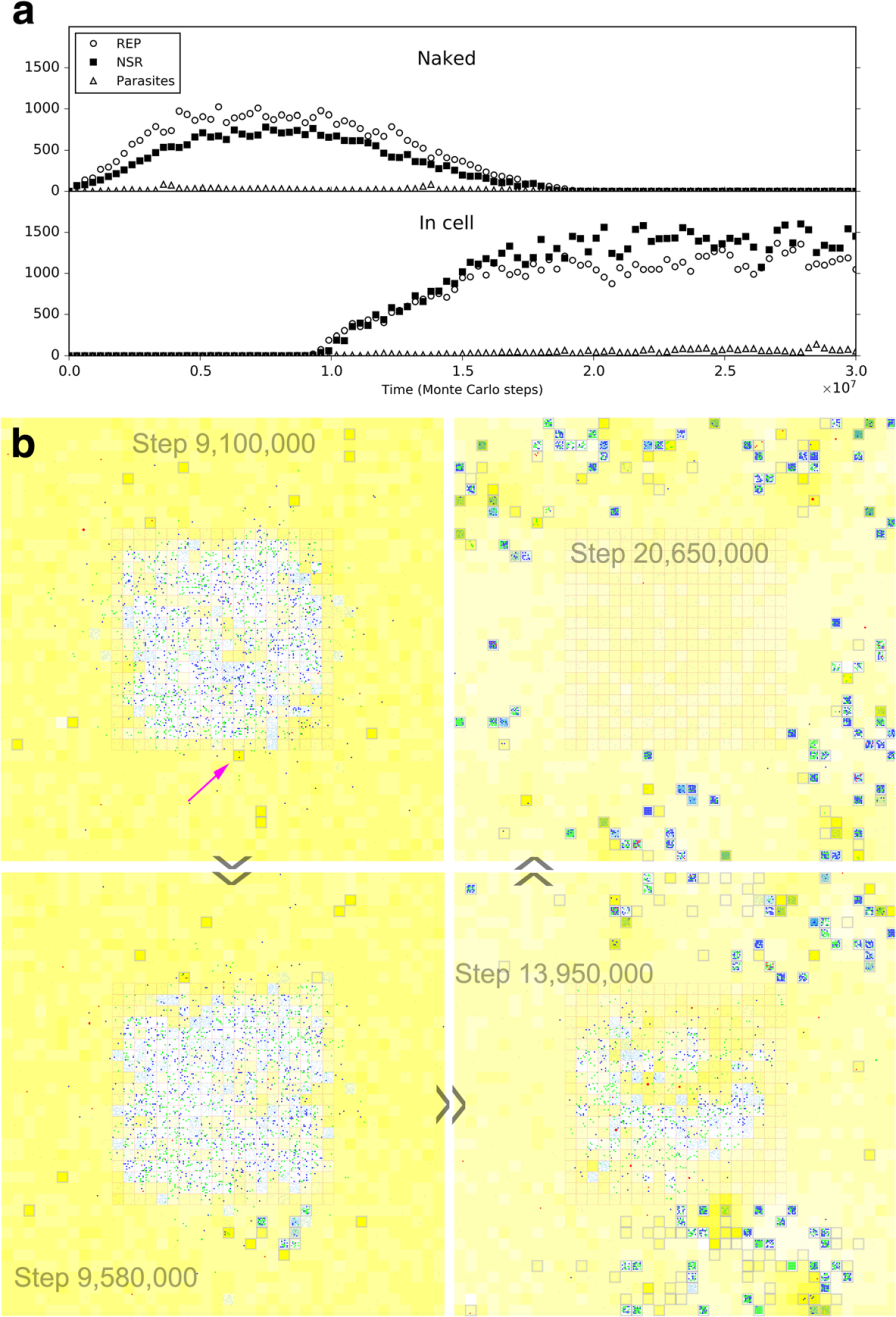
The natural emergence of protocells from the naked scene with REP and NSR thriving**.** The difference of this case from the case of Fig. 9 is that the protocells which are inoculated at step 6 × 10^6^ are empty ones, and the rate of cytophagy is magnified by 100-fold (i.e., *P*_CC_ = 1× 10^− 5^). **a** The dynamic plot. **b** Snapshots showing the appearance of the first protocell containing the two ribozymes (arrow) by cytophagy at the rocky region’s edge and the subsequent spread of such protocells in the solution region (note that the grid has a toroidal topology). Meanwhile, naked REP and NSR in the rocky region decrease and finally disappear. The half-transparent frame at the edge of a grid room represents the protocell membrane. Other symbols have the same meanings as the ones in Fig. 6b

So then, it becomes attractive to modeling the natural emergence of these RNA-based protocells. In the system, after the initial inoculation of amphiphile precursors, they may transform to amphiphiles with probability *P*_AF_. Due to random movement of the amphiphiles, some location (i.e., a grid room) may happen to gather sufficient quantity (*L*_AM_) of such molecules to form a membrane (*P*_MF_). However, this is also a rare event determined by chance. So we inoculate several empty protocells a period after the initial step (the case shown in Fig. 10) (just like our motive for inoculating REP in the naked scene). The protocells may attract amphiphiles, grow and perhaps divide if the molecular number on the membrane rises to twice the *L*_AM_. In addition, during the process, there may be quite a few other events associated with the protocell (e.g., amphiphile leaving the membrane, protocell’s breaking, protocells’ fusion, etc.— for details, refer to relevant parameters listed in Table 1, and see Fig. 1 and Methods). Finally, the number of these empty protocells could reach a balance (also see our previous studies [36, 39] for more detailed analysis on such processes).

In the scene we assume here, obviously, we may expect empty vesicles, if formed in the solution, might move to the edge of the rocky region and absorb or engulf the ribozymes which is thriving in the rocky region. Therefore, based upon the thriving of REP and NSR in the rocky region, as well as the balance of empty vesicles in the surrounding solution system, we monitored the system to see what would happen. However, again, we experienced the difficulty to observe such “*de novo* incidents” which involves various random events. To facilitate the incident, we enhanced the rate of protocell’s cytophagy (*P*_CC_) to improve the empty vesicles’ chance to absorb the ribozymes (in reality, if the cytophagy rate is low, more time may be needed for such an incident to occur). As a result, we successfully observed the natural, de novo emergence of such RNA-based protocells, and the corresponding fading out of the naked phase (Fig. 10). Notably, in the modeling case, the observed cytophagy indeed occurs at the edge of the rocky region. The reason is that when the ribozymes diffuse to the liquid zone, they tend to move away (be “diluted”) and thus the chance for the formation of the RNA-based protocells would be little, especially considering that the resulted protocells should include both the two kinds of ribozymes. Such a case (Fig. 10) may be read as a demonstration for the plausibility of the emergence of protocells this way in reality. Remarkably, this is another case representing an evolution towards a more thriving living world (like the case of NSR emerging in a REP-thriving system, as mentioned above) – the number of the ribozymes within protocells, as a whole, become obviously greater than that of the naked ribozymes in the previous scene (Fig. 10a), while the raw materials are exploited to a greater extent (Fig. 10b – note the change of the color depth of the background yellow, which represents the quantity of raw materials, averaged in a system-wide scale).

Then, as planned, we traced into the process, to examine the detailed mechanisms concerning the superiority (and limitation) of the protocells in comparison with the naked phase. In fact, on account of the advantage of modeling studies, such explorations are, to some extent, convenient; and this in-depth analysis, as we will see, would greatly contribute to our understanding on the phenomenon concerning the emergence of cell-like form in life’s history.

### About evolutionary mechanisms behind the emergence of cellular form

From the case shown in Fig. 9, we see that parasites may make the naked subsystem unstable; on the other hand, because the protocells were inoculated before the naked subsystem encounters significant invasion of parasites, the victory of protocells should have other reasons or mechanisms (also see Fig. 10a, in which the natural arising of protocells occurs before the naked subsystem is threatened by parasites). To avoid the interference of parasites, which occurs by chance – mainly due to mutation in the ribozymes’ replication, we turn off the mutation events (i.e., *P*_FP_ was set to 0) when exploring the advantages and disadvantages of the cellular form in the following. Wherein, to explore the influence of the parasite problem itself, we inoculate parasites into the system manually (ad hoc).

**Fig. 11:**
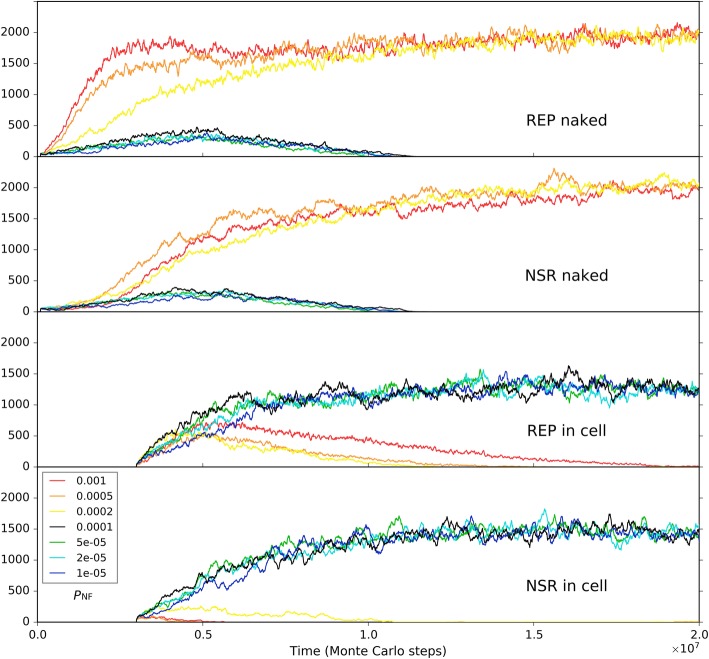
The influence of non-enzymatic nucleotide-synthesis rate (in regard of the cooperation of REP and NSR) on the competition between the protocell subsystem and the naked subsystem. Both subsystems adopt the default parameter values, except *P*_NPP_ = 1. Only raw materials (nucleotide precursors) were allowed to pass through the interface of the two subsystems. Similar to the case of Fig. 9, naked REP molecules and NSR molecules are inoculated at step 1 × 10^5^, but protocells are inoculated at step 3× 10^6^ (instead of step 6 × 10^6^). We can see that when *P*_NF_ is high, which means cooperation of REP and NSR is not important, the naked ribozymes in the rocky region achieve superiority; when *P*_NF_ is low, which means cooperation of REP and NSR is important, the protocells (containing the ribozymes) in the solution region achieve superiority. Notably, the change of *P*_NF_ may have distinct effects on REP and NSR (e.g., see the different distribution of red, orange and yellow lines between the “REP naked” and “NSR naked” subfigures, and that between the “REP in cell” and “NSR in cell” subfigures), which is not surprising according to the analysis above concerning *P*_NF_ (Fig. 5)

Firstly, to explore solely the effect of the membrane keeping the cooperative ribozymes together, we removed all the differences on the parameter settings between the naked subsystem (the rocky region) and the protocell subsystem (the solution region), and the probability of raw materials permeating through a membrane (*P*_NPP_) was assumed to be 1. In other words, the protocells are only distinctive in respect of its membrane’s effect which ensures the spatial proximity of REP and NSR and thus their cooperation (in particular, the nucleotides synthesized by NSR would not leak out as well). Besides, to see a “fair competition” between the two subsystems, only raw materials (nucleotide precursors) were allowed to pass through the two subsystems’ interface (note: in the near-reality simulation, e.g., the cases in Figs. 9 and 10, nucleotides and RNA are also allowed to transfer across the rocky edge). The analysis concerning the membrane’s significance for the ribozymes’ cooperation was performed by changing the rate of non-enzymatic nucleotide synthesis, *P*_NF_. As mentioned above, *P*_NF_ is a key parameter determining the cooperative relationship of the two ribozymes: when *P*_NF_ is low, NSR becomes important and the cooperation of the two ribozymes is evident (Fig. 5). Here we found that a lower *P*_NF_ may favor the protocell subsystem, whereas a higher *P*_NF_ may favor the naked subsystem (Fig. 11). In other words, when cooperation is significant, the strategy of encompassing the two ribozymes together would become effective; on the contrary, when cooperation is not so significant, a free mode for the ribozymes may even be better.

**Fig. 12:**
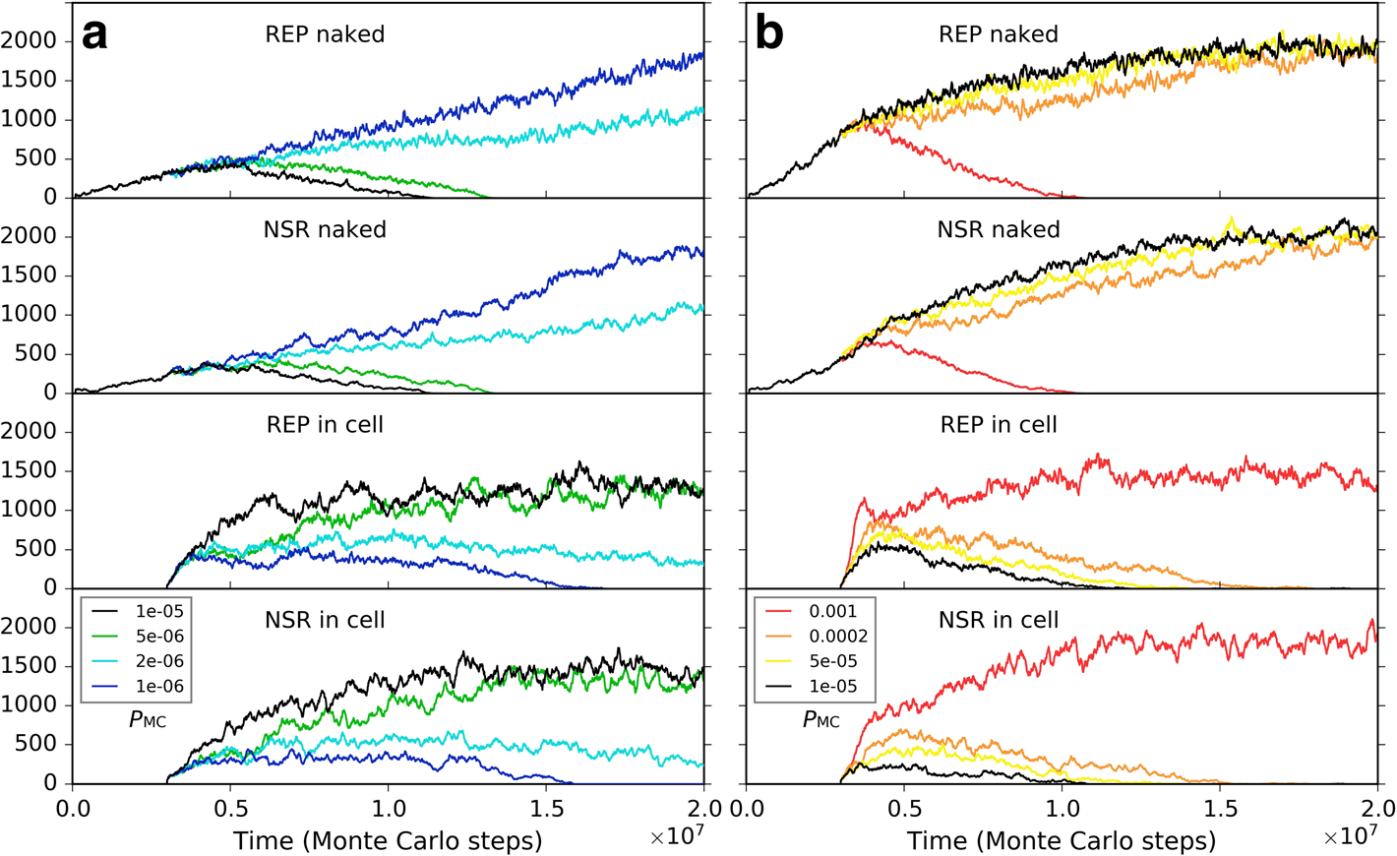
The influence of protocells’ moving rate on the competition between the protocell subsystem and the naked subsystem. **a** Black line (*P*_MC_ = 1 × 10^− 5^) corresponds to the case of black line in Fig. 11, where *P*_NF_ = 0.0001 and the protocells achieve superiority. When *P*_MC_ is turned down here, the protocells lose the superiority. **b** Black line (*P*_MC_ = 1 × 10^− 5^) corresponds to the case of yellow line in Fig. 11, where *P*_NF_ = 0.0002 and the naked ribozymes have the superiority. When *P*_MC_ is turned up here, the situation reverses

In the analysis above about the influence of molecular movement, it has become clear that the moving rate of the ribozymes may influence their spread (e.g., Fig. 7c), which can be related to the ribozymes’ accessibility to raw materials. So we supposed that the moving rate of the protocells, which is associated with the cellular ribozymes’ accessibility to raw materials, may also have a significant influence on the competition between the two subsystems here. We do such an analysis based upon two of the cases investigated above (Fig. 11). We found that for a case in favor of the spread of protocells, if *P*_MC_ is turned down, the protocells may lose their superiority (Fig. 12a); whereas for a case in which the protocells are disfavored, if *P*_MC_ is turned up, they may gain superiority (Fig. 12b). As an implication here, we see that a more active motion of protocells may bring more chance for those ribozymes within them to access raw materials all around the environment. Indeed, the membrane separates the ribozymes from the environment, providing them with an opportunity – while being held together for cooperation, able to search around the environment for raw materials. As an analogue in modern living world, active motion is very significant for heterotrophic organisms (e.g., protozoa and animals).

**Fig. 13:**
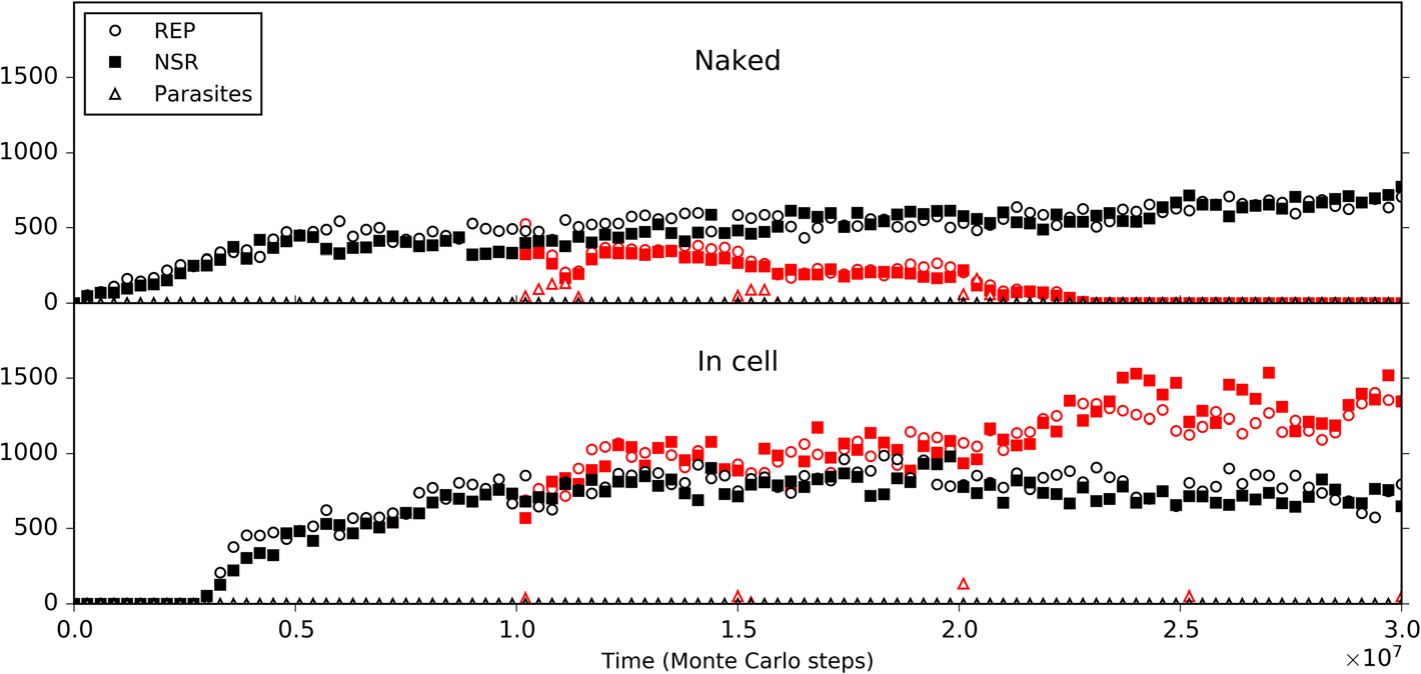
The influence of parasites on the competition between the protocell subsystem and the naked subsystem. Based upon the studies above (Figs. 11 and 12), by adjusting parameter values of *P*_NF_ and *P*_MC_, as well as testing different random seeds, we managed to find a case in which the two subsystems are almost at balance (represents by black symbols; *P*_NF_ = 0.0001 and *P*_MC_ = 5 × 10^6^). The situation is actually a little in favor of the naked ribozymes. After step 1 × 10^7^, 50 molecules of parasites with a sequence of “*CS*_Tag_ + *CS*_Tag_” (triangles) are inoculated into the two subsystems simultaneously (for the naked subsystem, distributed in 10 randomly chosen grid rooms containing REP and NSR, five each; for the protocell subsystem, distributed in 10 randomly chosen protocells containing REP and NSR, five each) every 5 × 10^6^ step, resulting in the victory of protocells (see red symbols)

To assess the protocell’s superiority to naked ribozymes in the rocky region in respect of resisting parasites, we attempted to choose a case in which the two subsystems is at balance, and then inoculate parasites into the subsystems in parallel to observe the outcome. However, we found that it is somehow difficult to obtain such a balancing case. Perhaps the two subsystems are basically not able to co-exist. No matter how, we managed to choose a “near-balance” case, but a bit in favor of the naked ribozymes (Fig. 13a). It was revealed that the tendency may be reversed on account of the periodically inoculation of parasites (due to the assumption *P*_FP_ = 0, as mentioned in the beginning of this section, parasites are here difficult to appear de novo in the system) – that is, the protocells win the competition finally (Fig. 13b). Note that the inoculation is administrated simultaneously and equally to the two subsystems (see the legend of Fig. 13). The result is easy to understand – parasites destroying a protocell are difficult to invade other protocells on account of the membrane, whereas in the naked region, locally thriving parasites are easy to move around and “attack” ribozymes elsewhere.

**Fig. 14:**
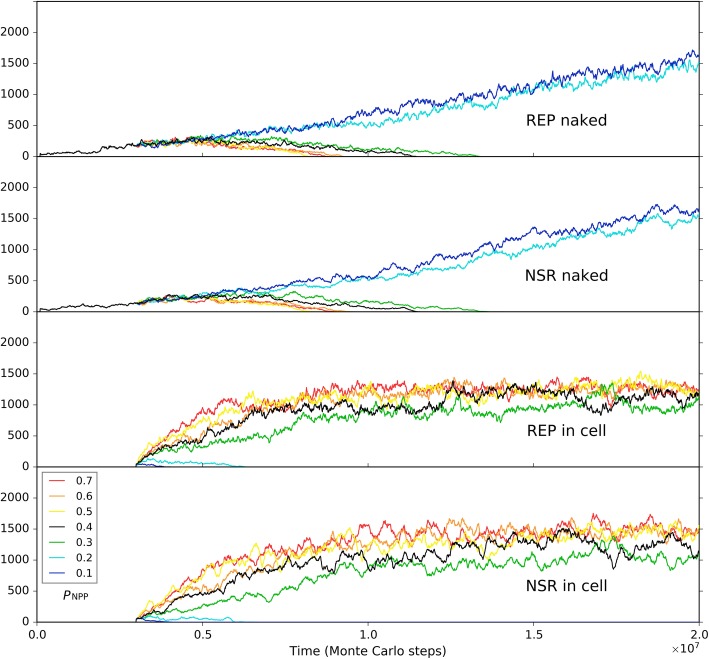
The influence of the membrane’s permeability on the competition between the protocell subsystem and the naked subsystem. The cases shown here adopt the same parameter values as the black-line case in Fig. 11, except *P*_NPP_ (for the black-line case in Fig. 11, *P*_NPP_ = 1)

Next, we turned to the influence of the membrane permeation. So far, this is perhaps the only obvious element we percept concerning the limitation of the cellular form. In reality, the permeability of raw materials to the membrane can certainly not be 100% (i.e., *P*_NPP_ = 1, as assumed above, cannot occur). Such a permeability may have been crucial for natural selection to “assess” the protocells’ overall advantage. Indeed, our simulation showed that the superiority of the protocells is significantly influenced by this factor (Fig. 14). When *P*_NPP_ is high, the protocells overwhelm the naked ribozymes in the rocky region; whereas when *P*_NPP_ is low to some extent (here equal to or below 0.2), the protocells may lose the competition. That is, a low permeation rate may reverse the superiority of the protocells.

**Fig. 15:**
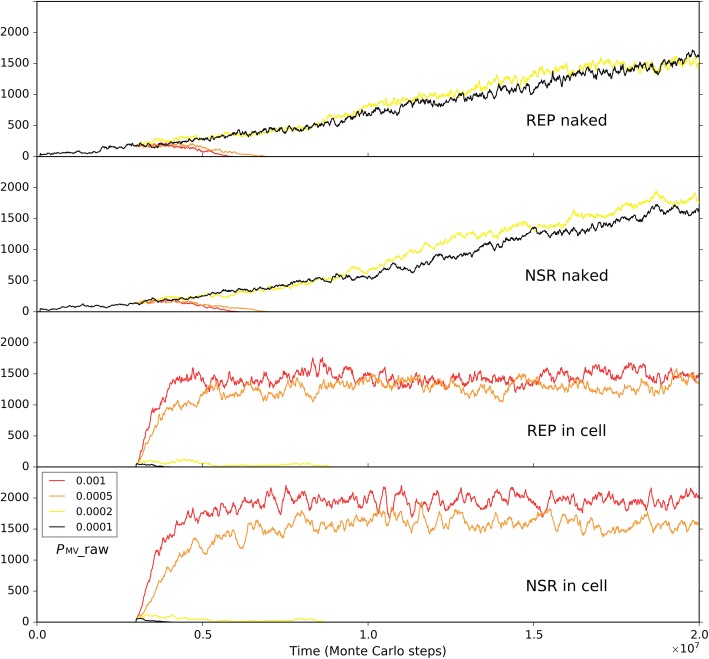
The influence of the moving rate of raw materials in the solution on the competition between the protocell subsystem and the naked subsystem. The black-line case is the same as the blue-line case in Fig. 14. When the moving rate of raw materials in the solution region is increased to 0.0002 (the yellow line), both the ribozymes within protocells in the solution region and the naked ribozymes in the rocky region are benefited due to their greater accessibility to the raw materials (the naked ribozymes in the rocky region are also benefited because raw materials in the solution region may diffuse into the rocky region). However, when the moving rate of raw materials in the solution region increases further (the orange and red lines), the ribozymes within protocells (or say, the protocell subsystem) may win the superiority because they, no matter how, benefit from this “condition change” more directly

Finally, we should consider a factor associated with the environment the protocells may confront in reality, though it appears not a feature of the protocells in themselves. We noticed that for the blue-line case in Fig. 14 (*P*_NPP_ = 0.1), when judging from the three key parameters mentioned already (*P*_NPP_, *P*_NF_, *P*_MC_), the condition is the same as the near-reality case (Figs. 9 and 10). Why the protocells lose the competition here but achieve superiority there? Considering the difference between the two cases, we threw our suspicion on that factor: the moving rate of raw materials – in the “reality case”, the moving rate of raw materials in the solution is much higher. Remarkably, besides the moving rate of protocells (Fig. 12), this is the other factor in relation to the accessibility of raw materials, which is crucial for the thriving of ribozymes (see the section “About the movement of molecules”). Indeed, when we changed the moving rate of raw materials (in the solution region) to approach the level in the reality case, the balance of competition shifted toward the protocell dramatically (Fig. 15). In other words, this result suggests that the protocells’ opportunity of living in free water body, where the mobility of raw materials should be much greater than in the rocky region, may, at least to some extent, compensate their disadvantage with regard to the membrane as a barrier to raw materials. The key point is, the protocells are robust to this environment – they would hold the ribozymes together “for the sake of” their cooperation and simultaneously enjoy the great accessibility of raw materials which is brought about by the high molecular moving rate in free water.

To summarize, through a series of devised analyses by adjusting parameters in our model, we investigated the evolutionary mechanisms in regard of the emergence of the cellular form from five aspects: four advantages and one disadvantage. The first apparent advantage is the membrane boundary’s role to keep the cooperative ribozymes together. A second apparent advantage is the membrane boundary’s role to prevent the systematical invasion of parasites; that is, if the parasites cannot get through the membrane, their damage would be limited to one protocell once. There seem to be only one disadvantage: the accessibility of raw materials is blocked by the membrane. This shortcoming is also obvious – it can be so dramatic that other advantages may be overwhelmed. Fortunately, the membrane may save itself in the same respect: its robustness against water flow renders the protocells able to “live” in free water, which “provides” a higher molecular mobility and thus an enhanced accessibility of raw materials. Moreover, if the protocells may evolve the ability (in future) to move actively, their accessibility to raw materials would be augmented further. This is, at least, a “reserved advantage”.

## Discussion

First, we should have a comment on the statistics concerning our approach. For the “transient regime” during the evolution of the system, the outcome may be quite different with a different seed. The difference is mainly manifested as the time step (Monte-Carlo step) when the transition would occur, for example, as for the emergence of NSR in a REP-thriving system and for the emergence of protocells in a naked stage (by cytophagy). However, for a stable stage, the main feature is the quantity-level of relevant objects (e.g., the number of ribozymes, protocells and raw materials), which is not much influenced by the difference of random seeds. The reason is that after numerous sampling over a large number of Monte-Carlo steps (in fact, most probabilities are sampled on for many times within one step), the balance-level of the quantities is just a representation of “average” feature of the system. In addition, from the fluctuation of the dynamics curves, the “deviation” feature also appears to be quite straightforward. In other words, the major outcomes here are quite stable due to inherent statistics-included feature of the simulation approach. Therefore, we have not conducted ad hoc statistics studies on the results (which would be almost computation-insurmountable owing to the large-scale of the model system), relying solely on the appearance of the dynamics curves instead.

The focus of the present study is about the evolutionary dynamics concerning the emergence of REP-NSR-contained protocells from a naked scene with REP and NSR. We demonstrate the plausibility of such a transition (Figs. 9 and 10), and subsequently investigate the evolutionary mechanisms possibly involved in the transition (Figs. 11, 12, 13, 14 and 15). In fact, with a similar model, we have investigated the cooperation of REP and NSR in the naked scene, as well as their cooperation in the protocells [36]. A distinction is that the REP there [36] is a ligase-type one, unlike the REP here, which is a polymerase-type one (as mentioned already in the Introduction). Obviously, from the results there and those here, we know that this difference does not alter the outcome that REP and NSR can cooperate to thrive. The investigation in Ref. [36] focused on the plausibility of the cooperation of the ribozymes (for the protocell model there, even including a third ribozyme: amphiphile synthetase ribozyme) and a systematical analysis of the parameters’ influence on the modeling results. It did not show the plausibility of the natural emergence of one ribozyme after another (here, NSR after REP) and the plausibility of the natural emergence of the RNA-based protocells (by empty protocells’ cytophagy of ribozymes). More importantly, here we investigated the evolutionary mechanisms possibly involved in the transition from the naked stage to the cellular stage (Figs. 11, 12, 13, 14 and 15), which is absent in the previous study [36].

To envision a complete scenario – from the earliest molecular form, we start from the spread of REP alone, and then to the co-spread of REP and NSR in the naked scene (Figs. 2, 3, 4, 5, 6, 7 and 8). In fact, it turns out to be valuable to perform such a “prelude” study, which provides information about the way to judge the ribozyme’s cooperation (i.e., *P*_NF_ in this model), the different effects of moving rates regarding raw materials, ribozymes, and parasites, etc. – such information is important for us to examine possible advantages and disadvantages regarding the advent of the cellular form. But notably, as mentioned already, for the naked scene in reality, we do not insist REP should have emerged before NSR. That is to say, NSR may have emerged first (it would favor its own replication by supplying nucleotides) [31], replicating through non-enzymatic template-directed synthesis [35]. If so, the bottle-neck would then become the template-directed synthesis, and a selective pressure for the emergence of REP would be obvious. Certainly, when REP emerged, the story would all the same proceed into the naked scene with cooperative REP and NSR.

Alternatively, one may suggest that the transition from the molecular form to the cellular form may have occurred at the one-ribozyme stage, i.e., from REP to protocells containing REP (see [56] for a proposition about such a protocell as the simplest cellular life form), or from NSR to protocells containing NSR. Admittedly, we cannot rule out these possibilities. An apparent advantage for such transition is to resist parasites. In addition, notably, in replication, REP in itself need cooperation between the REP molecules as templates and those as catalysts; NSR, also, need cooperation, though not a direct one – nucleotides synthesized by NSR molecules as catalysts should be exploited by other NSR molecules as templates. The introduction of the membrane boundary may also facilitate such kinds of cooperation. However, a result in the present study suggests that when the cooperation between different types of ribozymes (REP and NSR) is not so important, a naked form might be better (Fig. 11). This is a sign shown that protocells may have emerged after the advent of at least two ribozymes. Additionally, considering the result here about the dramatic disadvantage of the membrane’s low permeability to raw materials (Fig. 14), the transition from REP to REP-contained protocells may be significantly impeded because nucleotides are difficult to permeate across a membrane [57, 58] (note that the transition from NSR to NSR-contained protocells would be less influenced in this respect because therein what need to pass through the membrane would be nucleotide precursors; see [18] for a more detailed discussion on relevant issues).

**Fig. 16:**
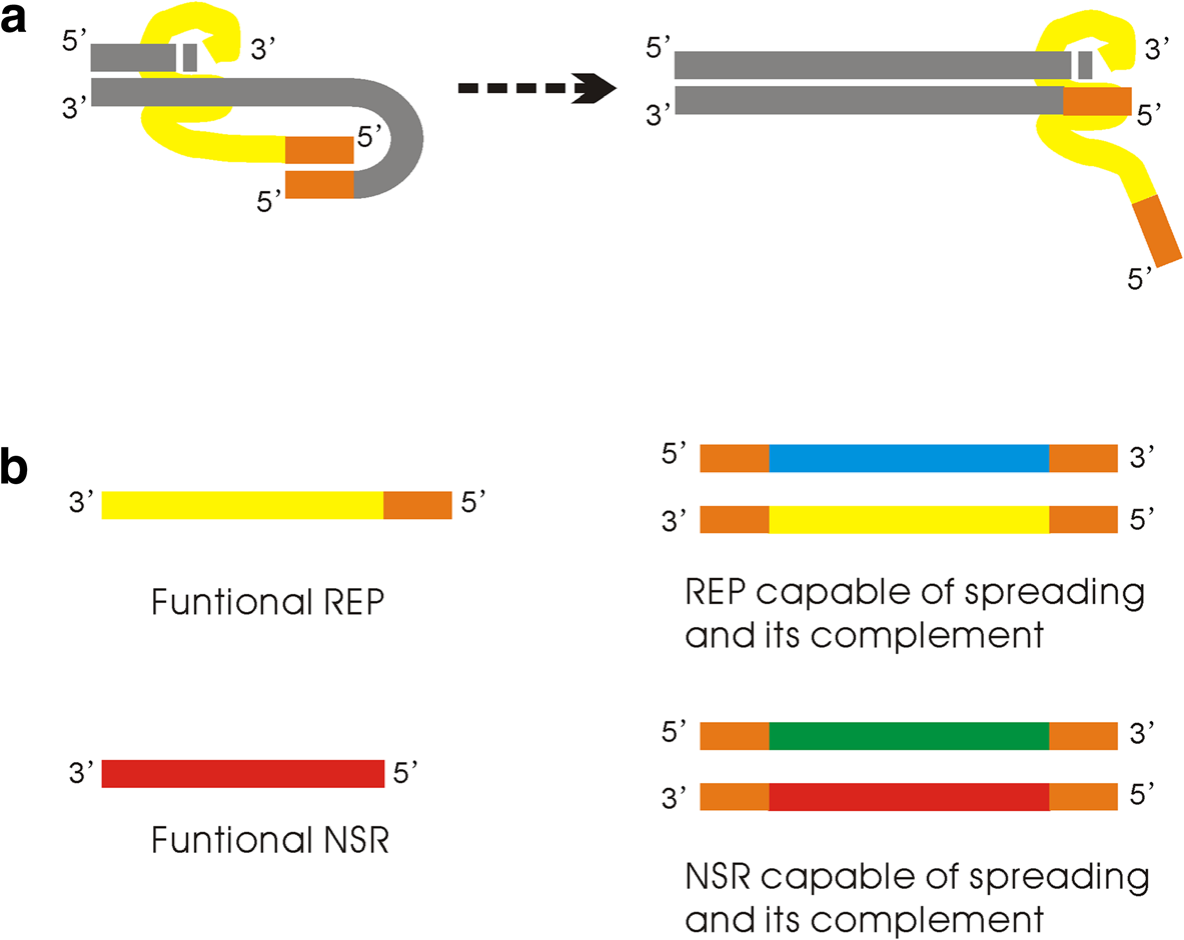
About the tag mechanism involved in the model. Color legends: (grey – template/substrate); (yellow – catalytic domain of REP); (orange – tag); (red – catalytic domain of NSR); (blue – complementary sequence of REP’s catalytic domain); (green – complementary sequence of NSR’s catalytic domain). Note that in a tag mechanism like this, the reverse-tag and the tag-recognizing domain of REP are actually of the same sequence as the tag (orange), which is palindromic. **a** REP binds onto the template by recognizing a 5′-tag, and when the copying proceeds to the tag segment, the base-pairing between the template’s tag and the REP’s tag-recognizing domain would separate. **b** A functional REP should contain a catalytic domain and a tag (actually a tag-recognizing domain), whereas a functional NSR needs only to contain its catalytic domain. To be able to spread in the system by replication, both REP and NSR must contain two tags flanking the catalytic domain (actually a 5′-tag and a 3′-reverse-tag)

In a previous study, we showed that a tag mechanism, in which the REP recognizes its template through a short subsequence, should be important for a polymerase-typed REP to spread in the system [43]. The REP adopted in the present model is just a tag-mediated polymerase-typed REP. It should be noted that the mode of the tag mechanism here is adopted in the light of relevant experimental work (see Methods, Fig. 16), which is somewhat different from the previous one [43]. However, the REP can spread in the system all the same, which implies the robustness of the tag mechanism as a strategy to favor the spread of a polymerase-typed REP. In addition, remarkably, it has been shown here that the tag mechanism can be extended to a multi-ribozyme system; that is, if the NSR contains the tag, it can also “make use of” the REP in its replication. Notably, in the model of Higgs’ group [37], it appears that no tag mechanism is needed, and this is on account of the difference between their model and ours. In our model, we have a resolution at the level of nucleotide residue – any other RNA species may exploit the REP and act as parasites. In other words, we adopt a model system more approaching to the reality, which reflects the more serious parasite problem in reality – and so thus can manifest the need for the tag mechanism.

No matter how, both our simulation and theirs come to a common conclusion on the effective cooperation of the two ribozymes, REP and NSR, in the naked scene. In fact, apart from the analysis in the Results section concerning Figs. 4 and 5, we can also read a sign of the cooperation directly from the scenario on the NSR’s emergence in a REP-thriving system (Fig. 6b). That is, though at the REP-alone stage, the REP molecules disperse well around the whole system (step 6,690,000), after the co-spread of REP and NSR, both the two ribozymes are limited spatially, distributed in an aggregative way (step 20,000,000; note that the grid has a toroidal topology – that is, those REP and NSR molecules around the left and the right edges, are actually gathering in the same region). Additionally, we can notice that raw materials (yellow background) elsewhere are not sufficiently exploited by the locally distributed ribozymes. With these visualized messages, we can feel directly that paradox: for the cooperation of the two ribozymes, the moving rate of molecules should not be too large; but for a greater accessibility to the raw materials around, a greater moving rate of molecules would be beneficial. So then, it is not surprising that the emergence of membrane boundary, which renders the ribozymes’ movement and the raw materials’ movement separable, would be welcome in the evolution. That is, the protocells can benefit from the faster mobility of raw materials in free water, enjoying a passively achieved accessibility of raw materials (Fig. 15), or even might move actively to exploit raw materials elsewhere (Fig. 12).

In fact, some other traditional theoretical studies, mainly based upon the concept of “replicator”, addressed problems related to the topic we considered here. One can refer to reviews in this area [3–5]. For example, there was work showing the scenario on the emergence of a REP-like replicator from a metabolically coupled replicator system (MCRS) [5, 59]. The MCRS comprises multiple functional replicators which cooperate to generate monomers, thus functioning as a whole like an NSR. As another example, there was work which deliberately compared the effect of the naked spatial limitation with that of the membrane compartmentalization on the evolutionary dynamics of REP-like replicators [60]. However, it is difficult to assess these results here, because they tend to work with models apparently more abstract, and sometimes adopted assumptions not so relevant to a real RNA world. More importantly, as far as we know, there are not yet such theoretical studies explicitly addressing the evolutionary dynamics involved in the transition from a naked molecular system to a protocell system.

For the concrete scenario we assumed here of the transition from naked REP and NSR to protocells containing REP and NSR, we envisage a porous rocky region with a solution region surround it, based upon the submarine-hydrothermal-vent hypothesis concerning the hatchery of life on the earth [53–55]. However, the modeling is generally valid for another two scenes regarding the naked stage, i.e., mineral surfaces [46–49] and lacunose ices [50–52]. For the latter, the model here can almost be entirely transplanted; for the former, a more accurate model should assume a solution region (the protocell subsystem) “suspended over” a mineral surface region (the naked subsystem).

Finally, what would occur next, after the emergence of protocells containing REP and NSR? Owing to the efficient spatial limitation provided by the membrane boundary, more ribozymes may have appeared and cooperated with the REP and NSR. For example, in a previous study we have shown the plausibility of the cooperation of an amphiphile synthetase ribozyme with REP and NSR within protocells (mentioned above already [36]). Other ribozymes, such as a nucleotide-precursor synthetase ribozyme, which enabled the RNA-based protocells to exploit more fundamental raw materials, may also have emerged. However, with the increase of the types of the ribozymes, the protocells would tend more to risk the loss of some “genes” during their division, which aroused the selection pressure for gene-linkage, i.e., for a chromosome [3, 61]. Interestingly, in a modeling study, we have demonstrated the plausibility of the emergence of a chromosome in RNA-based protocells [41]. In the model, the RNA chromosome is assumed to adopt a circular chain to avoid end-degradation, and thus can sustain a much greater length comparing with individual ribozymes; in addition, there are self-cleaving sites between the linked genes on the chromosome, which might give rise to individual ribozymes – thus solving the problem of the “gene expression” (see the original paper for a detailed discussion [41]). Here, it is worth noting that, now we know the tag mechanism may have been important for the spread of (polymerase-typed) REP [43], and would remain valid in a system comprising multiple ribozymes (REP and NSR) as shown in this study; a further attractive issue is, can the tags work in a context of chromosome which bears the linked genes? That is, may the tags have just represented some “preliminary promoters” – serving as control handles for the “gene expression”? If so, that would be a door open towards future for this primitive living world; from then on, the evolution towards complexity would become fully expectable, ultimately leading to the arising of life on our planet – that amazing thing.

## Conclusions

The transition from the molecular level to the cellular level, which is marked by the introduction of a membrane boundary, may have occurred naturally in early history of evolution. Indeed, such a membrane, which may self-assemble from amphiphilic molecules (e.g., fatty acids or phospholipids) in water, constitutes a straightforward way to ensure the cooperation of different genetic/functional molecules – thus, promising the subsequent evolution towards greater complexity and higher efficiency. Just as importantly, the membrane makes an effective obstacle to parasites (those molecules exploiting the functional molecules but offering no advantages). However, as the other side of the coin, the membrane would also impede the protocells to access raw materials for the synthesis of their own molecules – as a major disadvantage of the membrane. This shortcoming is expected to be compensated by another virtue of the cellular form (also associated with the accessibility to raw materials) – while guaranteeing the spatial proximity of the cooperative genetic/functional molecules inside, the entity as a whole is independent of the molecular movement in its environment. For instance, it may endure the impact of a fast molecular movement in free water, so as to achieve a greater accessibility of the raw materials therein; moreover, if the protocells could evolve the capability to move around actively (just like protozoa and some bacteria in our modern living world), they would be benefited further. Interesting, here, all these considerations have been validated and demonstrated vividly in a computer simulation study using a near-reality model with the RNA world scenario as a context. The present work represents a systematical exploration on the evolutionary dynamics of this critical transition.

## Methods

In a simulation case for the naked scene, nucleotide precursors of a quantity (*T*_NPB_; see Table 1 for the descriptions of parameters) are introduced at the initial step. In a simulation case for the scene involving protocells, besides nucleotide precursors, amphiphile precursors of a certain quantity (*T*_APB_) are introduced into the system as well. After the introduction of these initial substances, the system evolves according to the rules of the model, involving those various events. During the process of the simulation, various data may be monitored or recorded, including the number of different molecules, the number of protocells, the information about RNA’s chain-length in the system, the spatial distribution of molecules and protocells, etc.

### The setting of the parameters

The probabilities concerning the events in the system should be set according some rules. Reactions catalyzed by ribozymes should be much more efficient than corresponding non-enzymatic reactions, so *P*_TLR_> > *P*_TL_ and *P*_NFR_> > *P*_NF_. “Template-directed ligation” should be apparently more efficient than “random ligation”, so *P*_TL_> > *P*_RL_. Here, nucleotide residues within the chain are assumed to be unable to decay (they should be protected therein), whereas those at the end of the chain decay at a rate lower than that of free nucleotides, i.e., *P*_NDE_ < *P*_ND_. Amphiphiles within a membrane should be protected, so *P*_ADM_ < *P*_AD_. Because of the self-assembly feature of amphiphiles, *P*_MF_> > *P*_CB_ and *P*_AJM_> > *P*_ALM_. The movement of molecules should be easier than protocells, so *P*_MV_ > *P*_MC_. Other considerations may include: *P*_BB_ may be higher than *P*_RL_, but lower than *P*_NDE_, *P*_CC_ should be quite low, *P*_APP_ > *P*_NPP_, etc.

In consideration of the computational intensity, some parameters, such as “total nucleotide precursors introduced in the beginning” (*T*_NPB_), “total amphiphile precursors introduced in the beginning” (*T*_APB_), “the lower limit number of amphiphiles to form a membrane” (*L*_AM_), and the lengths of the characteristic domains of the ribozymes and the tag (*CS*_REP_, *CS*_NSR_ and *CS*_Tag_), are set obviously smaller in scale than the corresponding situations possible in reality. However, such simplifications are believed to be not in conflict with the fundamental rules involved in the target system which is modeled.

In the study, we set the parameters mainly based on our experience in previous studies using similar models [31, 36, 38–43]. The default values listed in Table 1 were only chosen out to shape the cases which are used to demonstrate our results (but see text in Results for interpretations when the default values are not used). In other words, the model is surely not strictly bound with these concrete values of the parameters. Actually, the outcome of the simulation turned out to be fairly robust against moderate adjustments of most of the parameters (also refer to the “Magnitudes” in Table 1). In addition, to be sure about the robustness of the results here, a typical case (for one parameter combination) has been repeated with at least ten different random seeds.

### Detailed mechanisms concerning how some of the parameters work

With the breaking of phosphodiester bonds, an RNA molecule may degrade into shorter ones (including nucleotides). When the breaking site of the chain is at a single-chain region, the breaking probability is *P*_BB_. When the breaking site is within a double-chain region, the two parallel bonds may break simultaneously, with the probability *P*_BB_^3/2^. The adoption of the index 3/2, instead of 2, corresponds to the consideration of the synergistic effect of the two breaking events.

The probability of the separation of the two strands of a duplex RNA is actually assumed to be *P*_SP_^*(r + 1)/2*^, where *r* is the number of base pairs in the duplex. When *r* = 1, the probability would be *P*_SP_. When *r* increases, the probability would decrease (because *P*_SP_, as a probability, has a value between 0 and 1). That is, the separation of the two strands would be more difficult if the base pairs are more. The introduction of the 1/2 corresponds to the consideration that self-folding of single chains may aid the dissociation of the duplex.

The probability of membrane formation is assumed to be 1-(1-*P*_MF_)^x^, where x equals to *a*-*L*_AM_ + 1, and *a* is the number of amphiphiles in the grid room. When *a* equals to *L*_AM_ (the lower limit of the number of amphiphiles to form a protocell membrane), the probability of membrane formation equals to *P*_MF_. This assumption concerns the consideration that the more amphiphiles there are in a grid room, the more probably they would assemble to form a vesicle.

The probability of an amphiphile leaving the membrane is assumed to be *P*_ALM_ / [1 + *F*_OP_ × *n*/(*b*/2)^3/2^], where *n* is the quantity of inner impermeable ions, including nucleotides and RNA (measured by the number of nucleotide residues), and *b* is the quantity of amphiphiles within the membrane. Wherein, *b*/2 (there are two layers in the membrane) is a “scale” representation of the surface area of the membrane. Consequently, (*b*/2)^3/2^ is a scale representation of the cellular space. Thus, *n*/(*b*/2)^3/2^ is a representation of the concentration of the ions. Further, *F*_OP_ × *n*/(*b*/2)^3/2^ represents the consideration for the “osmotic pressure effect”: a higher concentration of the inner impermeable ions would cause the protocell to be more swollen, and thus amphiphiles on the membrane are less likely to leave – this effect was demonstrated experimentally [62].

The probability of a nucleotide precursor permeating into a protocell is assumed to be *P*_NPP_ × (b/*L*_AM_) / [1 + *F*_DE_ × *n*/(*b*/2)^3/2^], where *n* is the quantity of inner impermeable ions and *b* is the quantity of amphiphiles within the membrane. The element (b/*L*_AM_) represents the consideration of the constraining effect of the cellular space on the influx of nucleotide precursors. That is, when *b* increases, meaning that the cellular space increases correspondingly, the probability of a nucleotide precursor permeating into the protocell would become greater. *F*_DE_ × *n*/(*b*/2)^3/2^ represents the consideration of the effect of Donnan’s equilibrium [63] – simply put, RNA and nucleotides, which are charged and impermeable, may suppress the incoming of permeable materials with the same charge, i.e., nucleotide precursors as it is assumed here (see [39] for a detailed explanation).

The probability of protocell division is assumed to be *P*_CD_ × (1–2 × *L*_AM_/*b*), where *b* is the quantity of amphiphiles within the membrane. When *b* is no more than twice that of *L*_AM_, the probability is no more than 0, i.e., the protocell could not divide. This assumption considers the fact that the larger the protocell, the more probably it would divide, on account of the physical instability.

The probability of the movement of an RNA molecule is assumed to be *P*_MV_/*m*^1/2^, where *m* is the mass of the RNA, relative to a nucleotide. This assumption represents the consideration of the effect of the molecular size on the molecular movement. The square root was adopted here according to the Zimm model, concerning the diffusion coefficient of polymer molecules in solution [64].

### Some detailed assumptions when implementing the model

In a time step, for a grid room, collision is checked for a certain number of times. Each time, RNA molecules (including nucleotides) in the grid room are grouped pairwise, simulating collision events. Upon a collision, one of the following three events may occur: the first, they may ligate end to end with each other; the second, if one of them is REP and the other contains a 5′-tag, the REP may bind onto the tagged RNA molecule (as a template); the third, the longer RNA molecule may act as a template and attract the shorter one (as a substrate) based on the rule of base-pairing.

Only unpaired nucleotide residues at the ends of an RNA chain (either at 3′- or 5′-end) may decay. Only when an RNA molecule reaches 5-nt length may it act as a template (generally in accordance to an experiment [65]). A bound polymerase ribozyme would drop from its template when the whole chain is copied, or may drop with *P*_RD_ when the copying has not yet been completed. The bound polymerase ribozyme on the template also risks chain-breaking with *P*_BB_. Certainly, when the ribozyme’s chain breaks, the resulting pieces would drop from the template. A single strand RNA would become a template when a REP binds onto it, or may turn to a template with *P*_RTT_ when attracting a substrate in the absence of REP.

In the light of recent experiments concerning the in vitro molecular evolution of RNA polymerase ribozymes [28, 29] (also refer to relevant studies in history [25]), we assume that the tag is at the 5′-end of the template and the REP contains a “tag-recognizing domain”, also at its 5′-end, which base-pairs with the template’s tag. For such a REP, actually, the tag and the tag-recognizing domain should be of the same sequence which is palindromic so that a REP molecule, acting as a ribozyme, can recognize another REP molecule, serving as a template. Furthermore, to guarantee its ability to spread (i.e., become thriving through replication) in the system, the REP should contain a “reverse-tag” at its 3′-end so that its complementary chain carries a 5′-tag, and can also be recognized. Obviously, here, the reverse-tag is also of the same sequence with the tag, owing to the tag’s palindrome. That is, in practice, a REP capable of spread in the system should contain its catalytic domain, plus two palindromic tag sequences at 3′- and 5′- end, respectively. Similarly, to be able to spread in the system, an NSR should also contain two tag sequences flanking its catalytic domain. The difference is that a functional REP (acting as a ribozyme) should contain its catalytic domain and a 5′-tag (as the tag-recognizing domain), whereas a functional NSR need only its catalytic domain. For example, in the cases shown in Results, we assume a 10-nt length for the catalytic domain of the ribozymes and 4-nt length for the tag. Therefore, a functional REP should be at least 14 nt long, and a REP capable of spreading should be at least 18 nt in length. A functional NSR should be at least 10 nt long, whereas an NSR capable of spreading should be at least 18 nt in length. See Fig. 16 for an illustration of the tag mechanism involved in the model.
